# Hypoxia induced LUCAT1/PTBP1 axis modulates cancer cell viability and chemotherapy response

**DOI:** 10.1186/s12943-019-1122-z

**Published:** 2020-01-21

**Authors:** Lin Huan, Tianan Guo, Yangjun Wu, Linguo Xu, Shenglin Huang, Ye Xu, Linhui Liang, Xianghuo He

**Affiliations:** 1 Fudan University Shanghai Cancer Center and Institutes of Biomedical Sciences, Shanghai Medical College, Fudan University, Shanghai, 200032 China; 2 Department of Colorectal Surgery, Fudan University Shanghai Cancer Center, Shanghai Medical College, Fudan University, Shanghai, 200032 China; 3 Key Laboratory of Breast Cancer in Shanghai, Fudan University Shanghai Cancer Center, Fudan University, Shanghai, 200032 China

**Keywords:** Hypoxia, lncRNA, LUCAT1, PTBP1, Alternative splicing, Chemoresistance

## Abstract

**Background:**

Hypoxic tumors are refractory to DNA damage drugs. However, the underlying mechanism has yet to be elucidated. We aimed to identify lncRNAs that upregulated under hypoxia and their effects on colorectal cancer (CRC).

**Methods:**

CRC cells were treated with 1% O_2_ to identify lncRNAs that upregulated under hypoxia. We integrated these lncRNAs with RNA-seq of 4 paired CRC tissues and TCGA data to get candidate lncRNAs. Multiple in vitro and in vivo assays were used to explore the role of LUCAT1 in CRC.

**Results:**

We identified a hypoxia-induced lncRNA LUCAT1 that facilitated the growth of CRC cells and contributed to drug resistance of CRC cells both in vitro and in vivo. Mechanically, LUCAT1 interacts with polypyrimidine tract binding protein 1 (PTBP1) in CRC cells, facilitates the association of a set of DNA damage related genes with PTBP1, thus resulting in altered alternative splicing of these genes. Moreover, ectopic expression of PTBP1 in CRC cells with knockdown of LUCAT1 abrogated the effects induced by LUCAT1 knockdown. Chemotherapeutics drug combined with LUCAT1 knockdown via antisense oligonucleotides (ASO) would get a better outcome in vivo, compared with group treated with chemotherapeutic drug only. Notably, LUCAT1 is upregulated in CRC tissues, compared to adjacent normal tissues; and CRC patients with higher LUCAT1 have a worse prognosis and poorly responded to chemotherapy in the clinic.

**Conclusions:**

Our data suggested CRC cells utilizes LUCAT1 to develop resistance to DNA damage drugs, and disrupting the LUCAT1/PTBP1 axis might be a promising therapeutic strategy for refractory hypoxic tumors.

## Background

Hypoxia is a common hallmark of solid tumors and contributes to the development and progression of many cancers [1]. Colorectal cancer (CRC) is the third common type of cancers and the leading cause of cancer-related death worldwide [2]. Like many solid tumors, hypoxic fractions existed in colorectal cancers [3]. Accumulating evidence demonstrates that many factors, such as hypoxia inducible factor 1 alpha (HIF-1α), are involved in survival, angiogenesis, invasion and metastasis of hypoxic tumor cell [4], and several inhibitors targeting hypoxic tumor cells have been developed [5]. However, hypoxic tumors are resistant to chemotherapy and are closely correlates with poor clinical outcomes. Thus, it is of particular importance to unveil new molecular mechanisms underlying refractory hypoxic tumors.

Long non-coding RNAs (lncRNAs) are greater than 200 nucleotides (nt) in length and cannot or hardly be translated into proteins. Increasing evidence demonstrates that many lncRNAs are aberrantly expressed across cancer types, and play key roles in cancer development and progression including malignant transformation, cell proliferation, survival, migration and genomic stability [6]. LncRNAs, such as miR31HG, linc-p21, linc-ROR, NEAT1, also participate in hypoxia signaling and favor tumor cells to acclimate the hypoxic microenvironment [7–10]. Despite this, the role of lncRNAs in hypoxia signaling, particularly in chemoresistance of hypoxic tumor, remains elusive.

Here we identified 25 lncRNAs that are induced by hypoxia and upregulated in CRC. Among them, hypoxic LUCAT1 could facilitate survival of CRC cells by suppressing DNA damage and apoptosis. LUCAT1 interacts with polypyrimidine tract binding protein 1 (PTBP1) and regulates the alternative splicing of its downstream target genes which are widely involved in cell growth and DNA damage. High LUCAT1 confers resistance to chemotherapeutic drugs in CRC cells. Patients with higher LUCAT1 expression have a worse prognosis and poorly response to chemotherapy in the clinic.

## Methods

### Cell culture

HEK-293 T, HCT-116, RKO, and LoVo cells were cultured in DMEM, McCoy’s 5A, RPMI-1640, and F-12 K medium respectively, supplemented with 10% fetal bovine serum and antibiotics at 37 °C in an atmosphere of 5% CO_2_. For hypoxia treatment, cells were cultured with 5% CO_2_, 1% O_2_ and 94% N_2_ or treated with 100 μM CoCl_2_ (Sigma-Aldrich, MO, USA).

To establish RKO cells resistant to Oxaliplatin (RKO-OXA), RKO cells were exposed to an initial Oxaliplatin concentration of 0.1 μM in RPMI-1640 supplemented with 10% fetal bovine serum and antibiotics for 2 week. The concentration of Oxaliplatin then sequentially increased to 0.5 μM (2 week), 1 μM (2 week), 1.5 μM (2 week), and 2 μM. RKO-OXA was maintained in 2 μM Oxaliplatin. The parental RKO cells (RKO-parental) were passaged in the same manner without Oxaliplatin treatment.

### Animal model

Six-week-old male NOD/SCID mice were housed in laminar flow cabinets under specific pathogen-free conditions with food and water provided ad libitum. A total of 3 × 10^6^ HCT-116 cells were subcutaneously injected into the right flank of the axilla of each NOD/SCID mouse. All subcutaneous tumors were collected and weighed after sacrifice. For western blot analysis, a small piece of fresh tumor tissue was put into RIPA lysis buffer (Beyotime, Shanghai, China) and crushed in a cryotube by shaking with a sterile steel ball. SDS-PAGE loading buffer was added into the samples and heated at 100 °C for 10 min.

To explore sensitivities to chemotherapeutic drugs, mice in the NC or LUCAT1 groups were randomly divided into three subsets, when tumors reached 5 mm in length. Each subset was intraperitoneally injected with either PBS, 5-FU (50 mg/kg) or Oxaliplatin (7.5 mg/kg) twice per week for 3 weeks.

In ASO (antisense oligonucleotides) and drug combination experiment, mice bearing HCT-116 tumors were randomly divided into three groups when tumors reached 5 mm in length, intraperitoneally injected with PBS or Oxaliplatin (5 mg/kg) and intratumorally injected with ASO-NC (5 nmol) (negative control) or ASO-LUCAT1 (5 nmol) twice per week for 3 weeks. The target sequence was 5′-GCCTGTACAGTTGTGTCCAA-3′, whose knockdown efficiency was validated in vitro.

Tumor volume was calculated using the formula (length×width^2^)/2. All subcutaneous tumors were collected and weighed after sacrifice. All animal studies were conducted in accordance with relevant guidelines and regulations and were approved by the Animal Care and Use Committee of Fudan University.

### Human tissues

We studied 4 cohorts of colorectal cancer tissues (Additional file 1: Table S1): 1) 4 CRC tissues with matched adjacent normal tissues for RNA-seq analysis; 2) 46 CRC tissues; 3) 97 CRC tissue with matched adjacent normal tissues; 4) Neoadjuvant chemotherapy (NACT) CRC tissues, 78 NACT CRC tissues with 71 matched adjacent normal tissues. CRC and adjacent tissues were collected from Fudan University Shanghai Cancer Center (Shanghai, China). Upon resection, tissues were either stored in RNAlater (Thermo Fisher Scientific, IL, USA) or frozen in liquid nitrogen. This study was approved by the Ethics Committee of Fudan University Shanghai Cancer Center. Written informed consent was obtained from each patient in accordance with institutional guidelines.

### The Cancer genome atlas (TCGA) data

The differential expressed lncRNAs in cohort COAD (Colon Adenocarcinoma) in TCGA was obtained from circlncRNAnet (app.cgu.edu.tw/circlnc/circlncRNAnet/) [11]. The survival plots of LUCAT1 in TCGA-COAD were generated from GEPIA (Gene Expression Profiling Interactive Analysis) [12]. 15 genes (ACOT7, ADM, ALDOA, CDKN3, ENO1, LDHA, MIF, MRPS17, NDRG1, P4HA1, PGAM1, SLC2A1, TPI1, TUBB6 and VEGFA) were chosen as hypoxia signature genes, which were defined based on gene function and meta-analysis of cancers [13]. We put these 15 genes into GEPIA as a signature and calculated correlations with each lncRNA. Drug response data of TCGA-COAD were obtained through the TCGA data portal.

### Oligonucleotide transfection

All siRNAs or negative control (siNC) were synthesized by Biotend (Shanghai, China). The sequences of all siRNAs are shown in Additional file 1: Table S2. Around 1~2 × 10^5^ cells were seeded in each well of a six-well plate, and in the next day cells in each well were transfected with 5 μL of siRNA or siNC (20 μM) using Lipofectamine RNAiMAX (Invitrogen, CA, USA) according to the manufacturer’s instructions. After 48 h, the cells were harvested for further experiments. For RNA-seq, RNA was extracted after 24 h.

### RNA isolation, quantitative PCR (qPCR) and reverse transcription PCR (RT-PCR)

RNA was extracted using Trizol reagent (Invitrogen, CA, USA) according to the manufacturer’s protocol. cDNA was synthesized by PrimeScript RT Reagent Kit (TaKaRa, Tokyo, Japan). SYBR Premix (TaKaRa, Tokyo, Japan) was used to detect the expression levels of genes of interest. And, the expression of the isoform with the exon skipped or the isoform with exon retained of each target gene was also determined by qPCR. The ratio of isoform with the exon skipped to isoform with exon retained represents the splicing of the gene. Quantitative primers used in this study are shown in Additional file 1: Table S3.

cDNA was synthesized by PrimeScript RT Reagent Kit using 500 ng total RNA. RT-PCR was used to detect splicing of the gene. RT-PCR primers used in this study are shown in Additional file 1: Table S4.

### Statistical analysis

The data are presented as the mean ± SEM of at least three experiments. General paired or unpaired t-tests and Chi-square tests were conducted using GraphPad Prism 7 (GraphPad Software, CA, USA), SPSS (IBM, NY, USA) or R (https://www.r-project.org/). IC50 was determined by GraphPad Prism 7 using nonlinear regression ‘dose response’. Pearson correlation was calculated by GraphPad Prism 7 or R. Statistical tests were justified as appropriate. *p*-values less than 0.05 were considered statistically significant (* *p* < 0.05, ** *p* < 0.01, and *** *p* < 0.001).

### RNA-seq and analysis

Total RNA was extracted from cells using Trizol. To screen the candidate lncRNAs, the RiboMinus Eukaryote Kit (Qiagen, CA, USA) was used to eliminate rRNAs, and the NEBNext Ultra Directional RNA Library Prep Kit (New England Biolabs, MA, USA) was used to build the RNA-seq library. Transcript expression was analyzed using StringTie (version 1.2.3) and quantified by FPKM (fragments per kilobase of exon per million fragments mapped).

For other analyses, an mRNA-seq library was constructed using VAHTS Stranded mRNA-seq Library Prep Kit (Vazyme Biotech, Jiangsu, China). Gene expression was analyzed using Hisat2 and was quantified by FPKM. Alternative Splicing Detector (version 1.2, Novel Bioinformatics, Shanghai, China) was used to identify alternative splicing events. Alternative Splicing Detector considers both altered junction reads and altered coverage of alternative splicing exons, and combined *p*-values less than 0.05 were considered as statistically significant alternative splicing events.

Gene annotation and analysis was conducted by metascape (http://metascape.org/).

Gene Set Enrichment Analysis (GSEA) was conducted according to the instructions from the Broad Institute using the preranked method. After gene expression was quantified by FPKM, log2 scaled fold change of all expressed genes was calculated between negative control group and knock down group, and imported into GSEA analysis. C2 (curated genesets), C5 (GO genesets), C6 (oncogenic signatures) and hallmark genesets from MSigDB (Molecular Signatures Database) were analyzed. Graphic representations of results were generated using the clusterProfiler package in R (https://www.r-project.org/).

Additional information is included in the Additional file 1.

## Results

### LUCAT1 is transcriptionally induced by HIF-1α under hypoxia

**Fig. 1:**
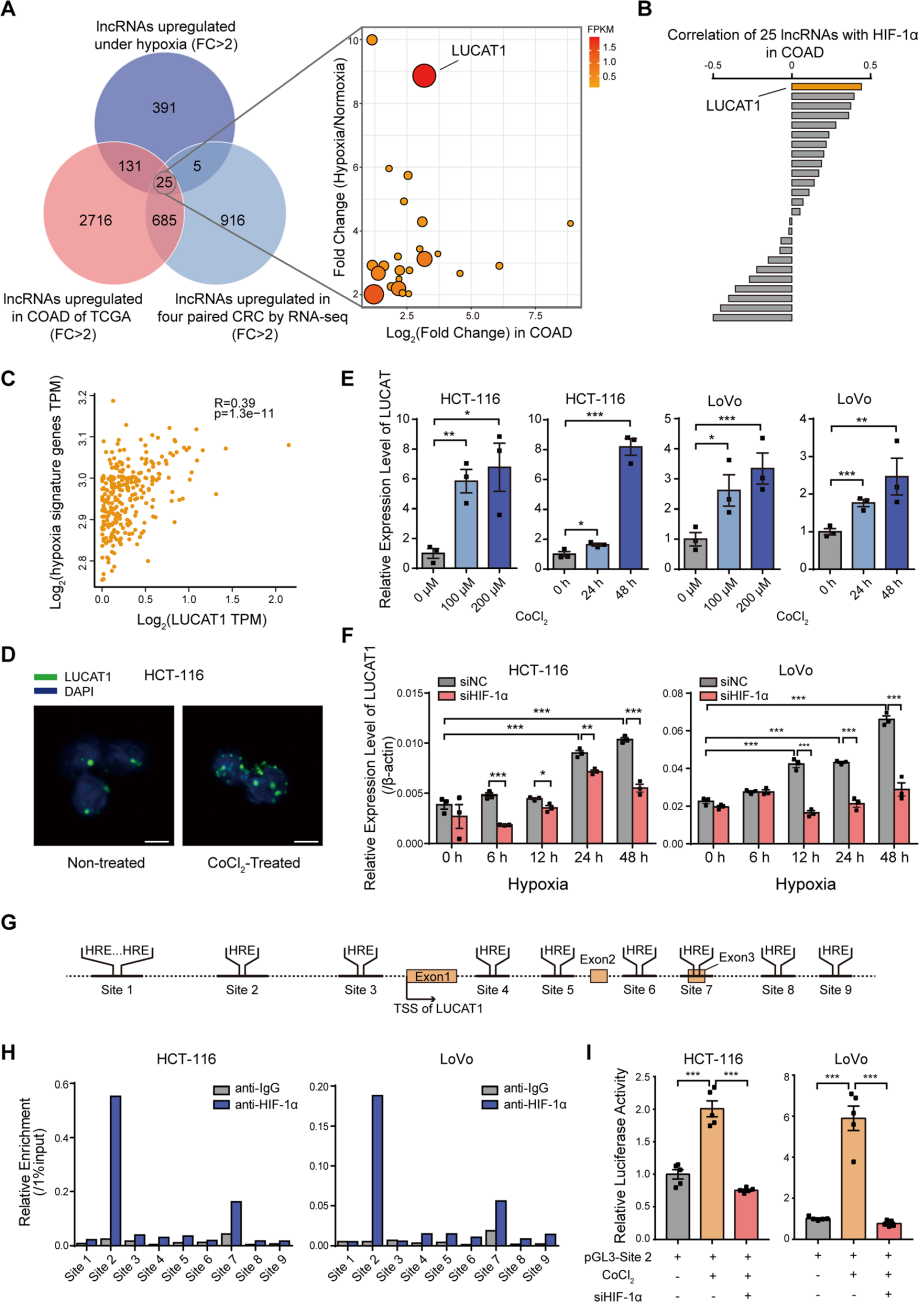
LUCAT1 is transcriptionally induced by HIF-1α under hypoxia. **a** Candidate lncRNAs were identified by RNA-seq of hypoxia treated RKO cell, RNA-seq of CRC tissues and TCGA dataset. **b** Correlation between HIF-1α and each candidate lncRNAs in TCGA dataset. **c** Correlation between hypoxia signature genes and LUCAT1 was generated from GEPIA. *n* = 275 CRC tissues in COAD. pearson correlation. **d** RNAscope assay of LUCAT1 localization in HCT-116 cells without or with CoCl_2_ treatment. **e** Relative LUCAT1 expression in HCT-116 and LoVo cells treated with CoCl_2_ was determined by qPCR. *n* = 3 independent experiments, two-tailed Student’s t-test. **f** qPCR was performed to determine relative LUCAT1 expression in HCT-116 and LoVo cells transfected with siNC or HIF-1α siRNAs under hypoxia at serial time points. *n* = 3 independent experiments, two-tailed Student’s t-test. **g** Schematic diagram of HREs in LUCAT1 locus. **h** ChIP assay demonstrating the binding capacity of HIF-1α to each HRE were conducted in HCT-116 and LoVo cells treated with hypoxia for 24 h. **i** Luciferase reporter assay for the second HRE activity of LUCAT1 in HCT-116 and LoVo cells with the indicated treatment. *n* = 5 independent experiments, two-tailed Student’s t-test. * *p* < 0.05, ** *p* < 0.01, and *** *p* < 0.001

To explore the role of hypoxia-induced lncRNAs in CRC, we first treated RKO cell with 1% O_2_ for 24 h, and found that 552 lncRNAs were upregulated (fold change> 2) under hypoxia. We then integrated these data with the differentially expressed lncRNAs (fold change> 2) in the cohort of COAD (Colon Adenocarcinoma) in TCGA (http://app.cgu.edu.tw/circlnc/) and in four paired CRC/ adjacent tissues sequenced by our group, and identified 25 hypoxia-induced lncRNAs that were upregulated in CRC tissues (Fig. 1a and Additional file 1: Table S5). We next analyzed the correlation of the level of hypoxia with the 25 lncRNAs. Of these lncRNAs, LUCAT1 was most highly correlated with HIF-1α (*R* = 0.4425) and hypoxia signature genes (*R* = 0.39) in TCGA-COAD (Fig. 1b, c and Additional file 1: Figure S1). LUCAT1, which is 890 nucleotide (nt) in length (NR_103548.1), is mostly located in the nucleus in CRC cells and has no coding potential (Additional file 1: Figure S2A-D). In situ fluorescence experiments also indicated that LUCAT1 was mostly located in the nucleus, and significantly upregulated in HCT-116 cells treated with CoCl_2_ (cobalt chloride) (Fig. 1d). We treated CRC cells with CoCl_2_, and found that CoCl_2_ induced LUCAT1 upregulation in a concentration- and time-dependent manner (Fig. 1e). Next, we investigated the correlation between LUCAT1 expression and HIF-1α expression in our CRC cohort. The results showed that LUCAT1 expression was also positively correlated with that of HIF-1α in our independent CRC cohort (Additional file 1: Figure S1E), which was consistent with the results from the COAD dataset. Moreover, the expression of LUCAT1 was significantly decreased upon HIF-1α knocked down under hypoxia (Fig. 1f and Additional file 1: Figure S2F). Conversely, the expression of LUCAT1 was increased after overexpressing a mutated HIF-1α, which lacks the oxygen-dependent degradation (ODD) domain and does not degrade under normoxia (Additional file 1: Figure S2G). To investigate whether LUCAT1 is a direct target of HIF-1α, we analyzed the characteristics of the genomic locus of LUCAT1, and found that many hypoxia response elements (HREs) located near LUCAT1 locus (Fig. 1g). ChIP assays revealed that HIF-1α mainly binds to the second HRE site (Fig. 1h). We then cloned the sequence around this HIF-1α binding site into a luciferase reporter construct. Luciferase activity was increased in cells treated with CoCl_2_ and was dramatically decreased in cells with HIF-1α-depletion (Fig. 1i and Additional file 1: Figure S2H). These results were consistent with the pattern of LUCAT1 expression in CRC cells under the same conditions.

### LUCAT1 inhibits DNA damage and apoptosis of CRC cells

**Fig. 2:**
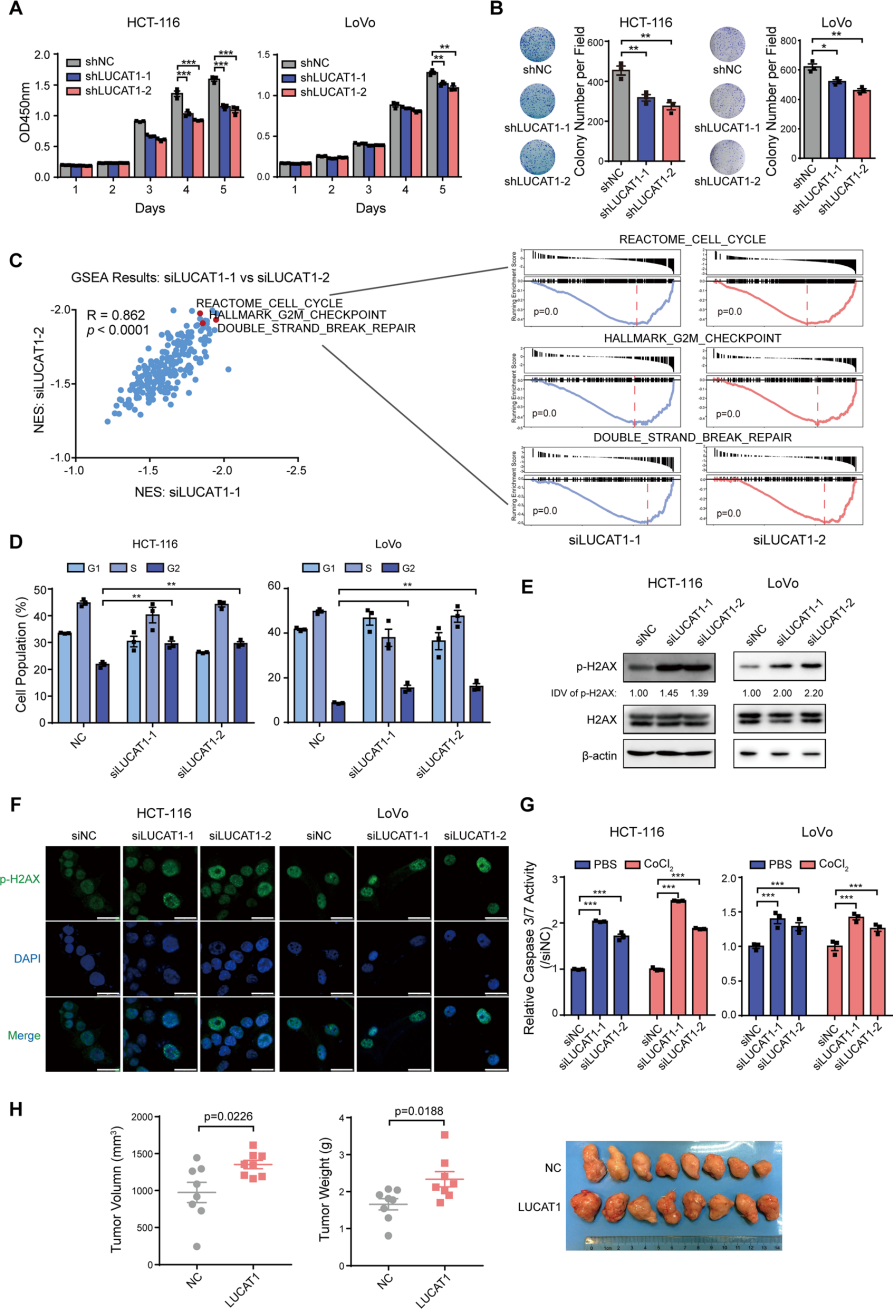
LUCAT1 inhibits DNA damage and apoptosis of CRC cells. **a** and **b** CCK8 and colony formation assays of HCT-116 and LoVo following NC or LUCAT1 knockdown. *n* = 3 independent experiments, two-tailed Student’s t-test. **c** GSEA analyses of RNA-seq data from HCT-116 cells transfected with siNC or LUCAT1 siRNAs. *n* = 180, pearson correlation. **d** Cell cycle analyses of HCT-116 and LoVo cells transfected with siNC or LUCAT1 siRNAs. *n* = 3 independent experiments, two-tailed Student’s t-test. **e** Protein expression levels of a DNA damage marker, p-H2AX, following NC or LUCAT1 knockdown as measured by western blotting. Integrated Density Value (IDV) was obtained by ImageJ and normalized by the first lane. **f** Confocal microscopic images of p-H2AX in HCT-116 and LoVo cells after NC or LUCAT1 knockdown. **g** Caspase 3/7 activities of HCT-116 and LoVo cells following NC or LUCAT1 knockdown without or with CoCl_2_ treatment. *n* = 3 independent experiments, two-tailed Student’s t-test. **h** Tumor volume and tumor weight of NC or LUCAT1 overexpressing HCT-116 cells in xenograft mouse model. *n* = 8 tumors, two-tailed Student’s t-test. * *p* < 0.05, ** *p* < 0.01, and *** *p* < 0.001

To determine the biological role of LUCAT1 in CRC, we first knocked down LUCAT1 expression in CRC cells and found the viability and colony formation capabilities of the cells were dramatically decreased (Fig 2a, b and Additional file 1: Figure S3A). Whereas, high expression of LUCAT1 in CRC cells infected with lentiviral construct containing LUCAT1 sequence significantly facilitated the viability and colony formation capabilities of the cells with and without CoCl_2_ treatment (Additional file 1: Figures. S3A-C). To explore the underlying signaling pathways involved in the biological effects of LUCAT1 in CRC cells, we conducted RNA-seq analyses in HCT-116 cells transfected with siRNAs against LUCAT1 (Additional file 1: Figure S3A). The results demonstrated that LUCAT1 participates in many biological processes related to cell growth, including DNA damage, cell cycle and G2/M checkpoint (Fig. 2c). Consistent with these results, G2/M arrests were observed in CRC cells when silencing LUCAT1 (Fig. 2d). Western blotting and in situ immunofluorescence assays demonstrated that the levels of serine 139 phosphorylated H2AX, a well-recognized marker of DNA double-stranded breaks [14], were dramatically increased after LUCAT1 knockdown (Fig. 2e, f, Additional file 1: Figure S4A and B). Additionally, the caspase 3/7 activities, which are often increased during apoptosis, were upregulated in the cells transfected with LUCAT1 siRNAs both with and without CoCl_2_ treatment (Fig. 2g). To examine the effect of LUCAT1 on CRC cell growth in vivo, HCT-116 cells overexpressing LUCAT1 were subcutaneously injected into the right flank of NOD/SCID mice. The results showed that tumor volume and weight were increased in the LUCAT1-overexpressing group compared to the control group (Fig. 2h). In addition, decreased DNA damage and increased proliferative marker were observed in tumors overexpressing LUCAT1 (Additional file 1: Figure S4C). These results indicated that LUCAT1 promotes CRC viability and the tumorigenicity by inhibiting DNA damage and apoptosis.

### LUCAT1 interacts with PTBP1 in CRC cells

**Fig. 3:**
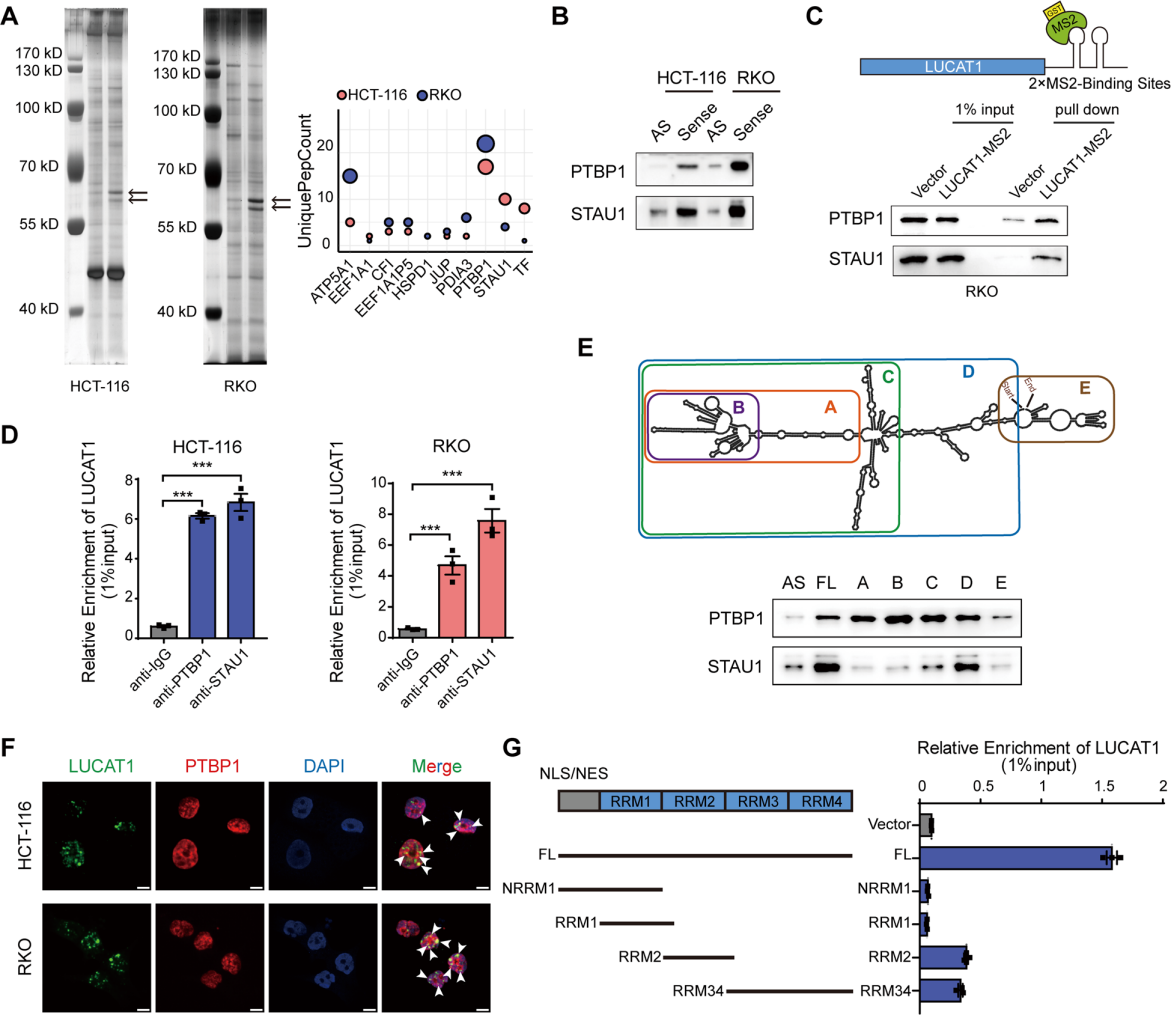
LUCAT1 interacts with PTBP1 in CRC cells. **a** Silver staining and mass spectrometry analyses following RNA pull down of LUCAT1 sense or LUCAT1 antisense in HCT-116 and RKO cells. **b** and **c** Western blotting validation of biotin-labelled RNA pull down using antisense or sense probes and GST pull down using empty vector or LUCAT1-MS2. **d** RIP assays were performed in HCT-116 and RKO cells. *n* = 3 independent experiments, two-tailed Student’s t-test. **e** Western blot of PTBP1 and STAU1 in biotinylated antisense, full length or truncated LUCAT1 pull down. **f** Colocalization of LUCAT1 and PTBP1 in CoCl_2_-treated HCT-116 and RKO cells. **g** Deletion mapping to identify the LUCAT1 binding domain in PTBP1 by RIP-qPCR using full length or truncated PTBP1 protein. * *p* < 0.05, ** *p* < 0.01, and *** *p* < 0.001

To explore the molecular mechanism by which LUCAT1 acts as an oncogenic lncRNA, we conducted biotin-labeled RNA pulldown assays followed by mass spectrometric analyses to search for the proteins that might interact with LUCAT1. Our results revealed several potential proteins that were pulled down with LUCAT1 RNA (Fig. 3a and Additional file 1: Table S6). Of these, PTBP1 and STAU1 (staufen double-stranded RNA binding protein 1) were the highest-ranked interactors. Further, PTBP1 is located in nucleus and STAU1 is partly located in nucleus, consistent with the localization of LUCAT1 in CRC cells. Biotin-labeled RNA pulldown and MS2-based GST pull down followed by western blotting analyses verified the interaction of LUCAT1 with PTBP1 or STAU1 (Fig. 3b, c and Additional file 1: Figure S5A). Furthermore, RIP assays showed that the antibodies against either PTBP1 or STAU1 could significantly enrich for LUCAT1 compared to controls (Figs. 3d, Additional file 1: Figure S5B and C). To determine the region of LUCAT1 that binds to PTBP1 or STAU1, a series of LUCAT1 fragments were generated based on the secondary structure predicted by RNAfold (http://rna.tbi.univie.ac.at/cgi-bin/RNAWebSuite/RNAfold.cgi) (Fig. 3e). The results demonstrated that STAU1 mainly binds to the fragments between the C and D of LUCAT1, whereas PTBP1 interacts with the majority of LUCAT1 except for the E fragment, consistent with the result predicted by RBPmap [15] (Fig. 3e and Additional file 1: Figure S6). Importantly, coincided with LUCAT1, PTBP1 dramatically influenced DNA damage, whereas STAU1 did not (Additional file 1: Figure S5D). Then we focused on PTBP1 as a candidate interacting partner of LUCAT1 for further investigation. PTBP1-CLIP (cross-linking and immunoprecipitation) data in starBase database [16, 17], further confirmed the interaction between LUCAT1 and PTBP1 (Additional file 1: Figure S5E). Interestingly, LUCAT1 were colocalized with PTBP1 in the nucleus of CoCl_2_-treated CRC cells (Fig. 3f). In addition, RIP assays using antibodies against Flag-tagged full-length or truncated PTBP1 were carried out and the results showed that the full-length PTBP1 protein is required for the strongest association with LUCAT1 (Fig. 3g and Additional file 1: Figure S5F). Taken together, these results indicated that PTBP1 is a bona fide interacting partner of LUCAT1 in CRC cells.

### LUCAT1 regulates mRNA alternative splicing through its association with PTBP1 in CRC cells

**Fig. 4:**
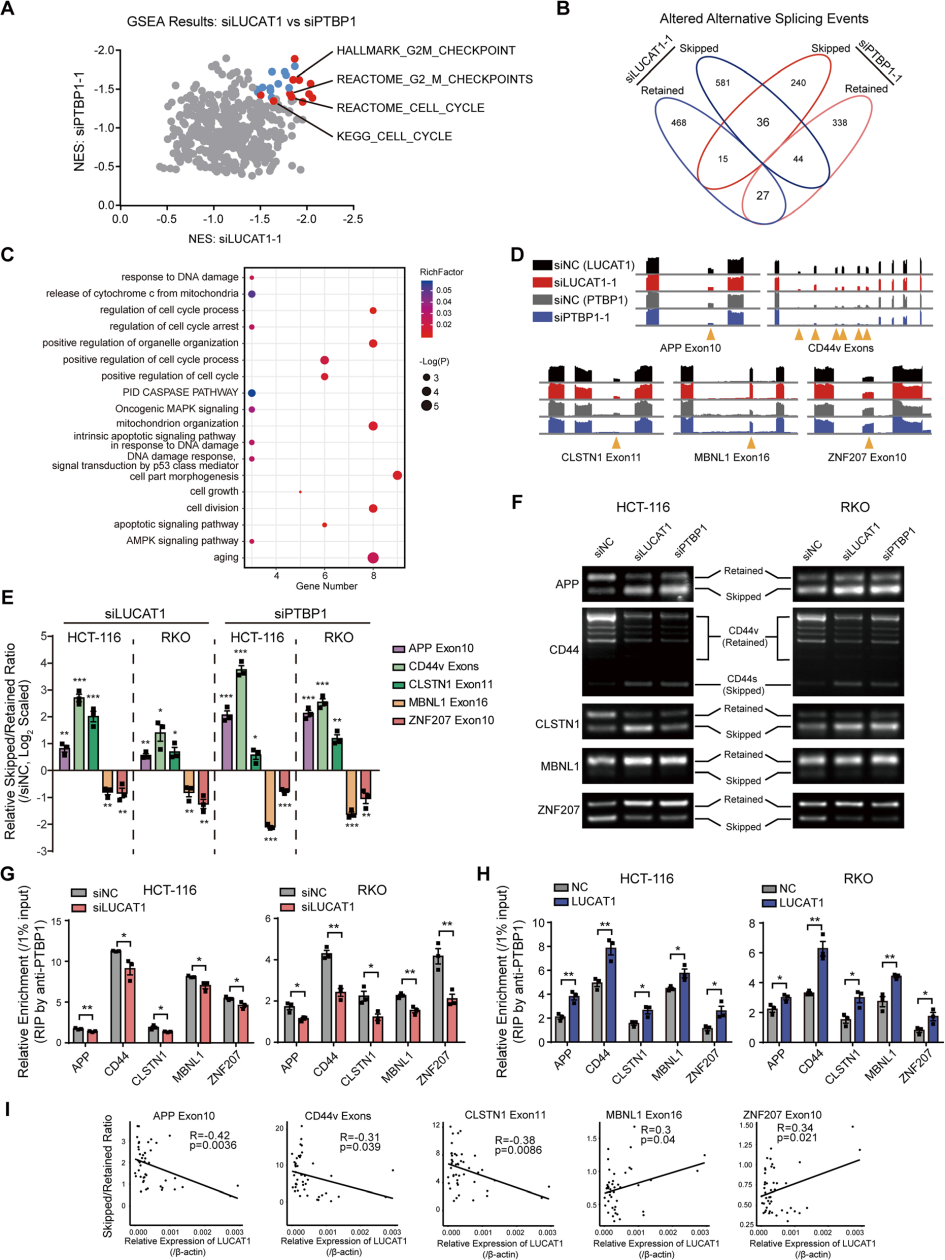
LUCAT1 modulates alternative splicing via interaction with PTBP1. **a** GSEA analyses of LUCAT1 knock down HCT-116 cells and PTBP1 knock down HCT-116 cells. Dots in colors are statistically significant. Dots in red are genesets involving cell growth, cell cycle and G2/M checkpoint. **b** Venn diagram of splicing events regulated by LUCAT1 and PTBP1. **c** Gene annotation and analysis of common genes alternatively spliced by LUCAT1 and PTBP1. **d** Exon coverage viewed in IGV. Arrows in orange indicates the corresponding exons. **e** Alternative splicing events in each target gene were determined by qPCR and normalized to siNC-transfected CRC cells. *n* = 3 independent experiments, two-tailed Student’s t-test. **f** Alternative splicing of target genes in CRC cells transfected with siNC, LUCAT1 siRNAs or PTBP1 siRNAs was validated by RT-PCR. **g** and **h** PTBP1-RIP assays were performed following LUCAT1 knockdown or overexpression. *n* = 3 independent experiments, two-tailed Student’s t-test. **i** Correlation between target gene alternative splicing and LUCAT1 expression in CRC tissues. *n* = 46 in CRC cohort, pearson correlation. * *p* < 0.05, ** *p* < 0.01, and *** *p* < 0.001

Next, we sought to determine the consequence of the interaction between LUCAT1 and PTBP1 in CRC cells. We first examined the expression and localization of PTBP1 following LUCAT1 overexpression. The results showed that neither LUCAT1 overexpression nor hypoxia affected the expression and subcellular distribution of PTBP1 in CRC cells (Additional file 1: Figures S7A and B). PTBP1 is an RNA binding protein that mainly participates in premature RNA splicing events and is associated with cancer progression [18]. Thus, we hypothesized that LUCAT1 overexpression would affect PTBP1-mediated alternative mRNA splicing. Intriguingly, the gene set enrichment analysis (GSEA) showed that the significantly enriched signatures upon LUCAT1 and PTBP1 knockdown are associated with cell growth, cell cycle and G2/M checkpoint (Fig. 4a and Additional file 1: Figure S8A). Most importantly, when we analyzed the alternative splicing events that were affected by LUCAT1 and PTBP1 knockdown, we found that LUCAT1 and PTBP1 regulated 63 common splicing events (36 skipped and 27 retained) in CRC cells (Fig. 4b). Gene Ontology (GO) enrichment by metascape showed these alternatively spliced genes are involved in the regulation of cell growth, cell cycle, apoptosis and DNA damage (Fig. 4c). Moreover, we used the Integrative Genomics Viewer (IGV) to identify the exon coverage of several genes associated with cell growth or DNA damage. The results showed that five representative genes (APP, CD44, CLSTN1, MBNL1, and ZNF207) had similar exon usage patterns in CRC cells following LUCAT1 or PTBP1 knockdown (Fig. 4d). We validated that the corresponding APP, CD44 and CLSTN1 exons were frequently skipped, while the sixteenth MBNL1 exon and the tenth ZNF207 exon were retained following knockdown of either LUCAT1 or PTBP1 in CRC cells, both by qPCR and RT-PCR analyses using specific designed primers (Fig. 4e, f and Additional file 1: Figure S8B-D). Furthermore, knockdown of these five genes significantly increased DNA damage and apoptosis of CRC cells both with and without CoCl_2_ treatment (Additional file 1: Figure S9A-E). To investigate how LUCAT1 influences alternative splicing events of these targets, we examined the ability of these genes to bind to PTBP1 after LUCAT1 overexpression or knockdown. Unlike LUCAT1, these five genes are mainly bind to the RRM2 or RRM34 domains of PTBP1 (Additional file 1: Figure S9F). RIP assays revealed that the binding of these mRNA targets to PTBP1 is decreased upon LUCAT1 knockdown, while PTBP1 binding to target mRNA is increased in CRC cells overexpressing LUCAT1 (Fig. 4g, h and Additional file 1: Figure S9G). Notably, these altered splicing events were correlated with the LUCAT1 expression in CRC tissues (Fig. 4i). Together, these results indicated that LUCAT1 modulates mRNA alternative splicing by influencing the binding of PTBP1 to its target genes.

### PTBP1 functions downstream of LUCAT1 in CRC under hypoxia

**Fig. 5:**
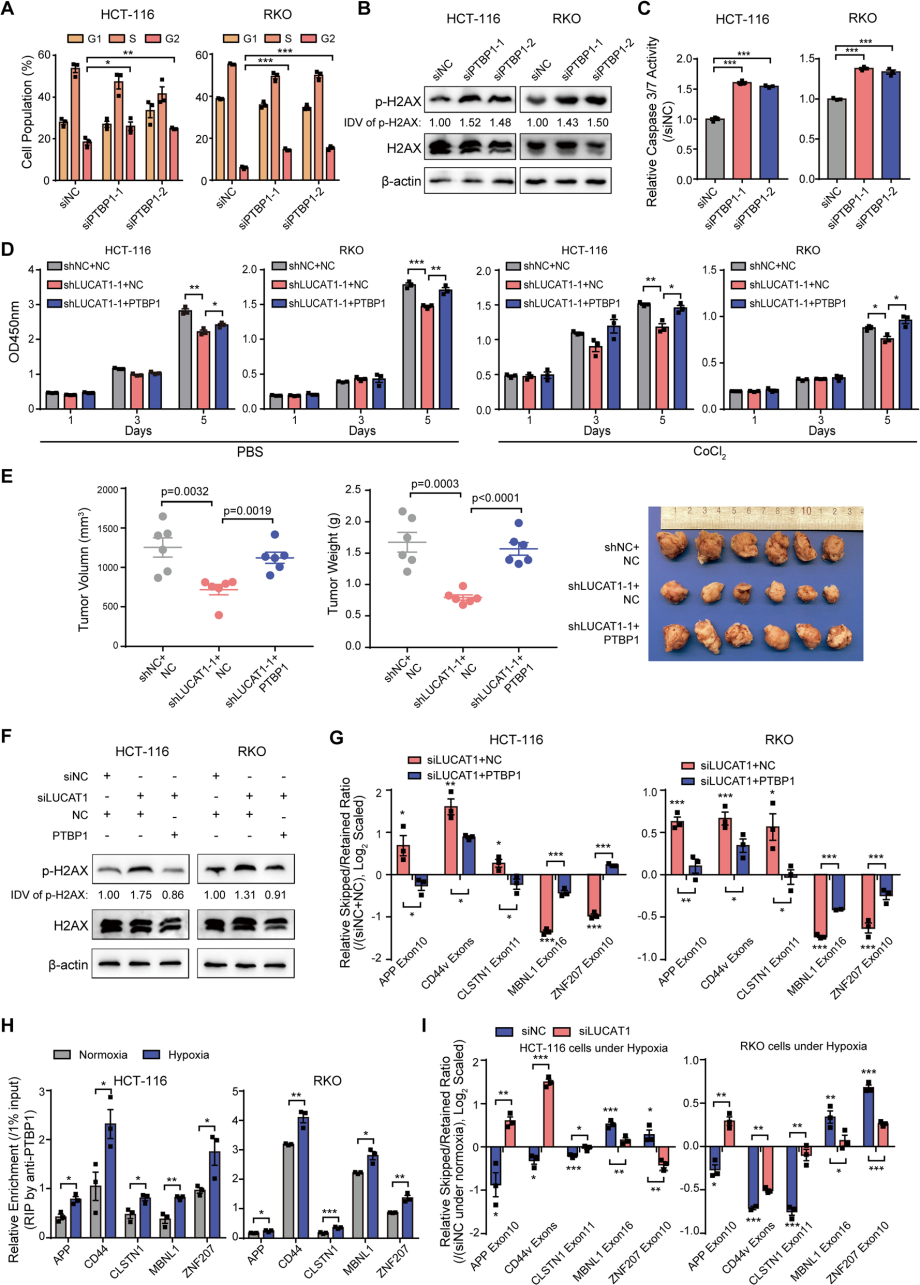
PTBP1 functions downstream of LUCAT1 in CRC under hypoxia. **a** Cell cycle (**b**) Western blotting for p-H2AX and (**c**) Caspase 3/7 activity assays in CRC cells transfected with siNC or PTBP1 siRNAs. *n* = 3 independent experiments, two-tailed Student’s t-test. Integrated Density Value (IDV) was obtained by ImageJ and normalized by the first lane. **d** CCK8 assays in LUCAT1-depleted CRC cells following reintroduction of PTBP1. *n* = 3 independent experiments, two-tailed Student’s t-test. **e** Tumor volume and tumor weight of indicated xenograft mouse model. *n* = 6 tumors, two-tailed Student’s t-test. **f** Western blotting for p-H2AX expression in LUCAT1-depleted CRC cells following reintroduction of PTBP1. Integrated Density Value (IDV) was obtained by ImageJ and normalized by the first lane. **g** qPCR assays of alternative splicing events in LUCAT1-depleted CRC cells following reintroduction of PTBP1. *n* = 3 independent experiments, two-tailed Student’s t-test. **h** RIP assays were performed in HCT-116 and RKO cells to evaluating the binding capacities between PTBP1 and target genes under normoxia or hypoxia. *n* = 3 independent experiments, two-tailed Student’s t-test. **i** Alternative splicing events in each target gene were determined by qPCR and normalized to siNC-transfected CRC cells under normoxia. *n* = 3 independent experiments, two-tailed Student’s t-test. * *p* < 0.05, ** *p* < 0.01, and *** *p* < 0.001

Previous reports have shown that PTBP1 acts as a tumor promoter in several cancers [19–21]. Our results also demonstrated that PTBP1 konckdown inhibits the viability and colony formation abilities of CRC cells; whereas PTBP1 overexpression promoted the viability and colony formation abilities of CRC cells (Additional file 1: Figures S10A-C). Notably, knockdown of PTBP1 also induced G2/M phase arrest, DNA damage and apoptosis which phenocopied the effects of LUCAT1 knockdown in CRC cells (Fig. 5a-c and Additional file 1: Figure S11A). To determine whether PTBP1 is a functional effector of LUCAT1, we reintroduced PTBP1 into CRC cells with LUCAT1 knockdown (Additional file 1: Figure S11B and C). The results showed that knockdown of LUCAT1 significantly inhibited the growth of CRC cells and induced DNA damage in CRC cells in vitro and in vivo, altered alternative splicing events, while reintroduction of PTBP1 abrogated the effects induced by LUCAT1 knockdown (Fig. 5d-g and Additional file 1: Figure S12A-C). Moreover, the suppression effect of LUCAT1 knockdown on CRC cell growth is reduced in PTBP1 knockdown cells than that in control cells (Additional file 1: Figure S13A and B).

Next, we investigated whether the LUCAT1/PTBP1 axis functions in CRC cells under hypoxia. We observed that the binding of the five target genes to PTBP1 increased under hypoxia (Figs. 5h and Additional file 1: Figure S14A). Moreover, these five genes showed similar patterns of alternative splicing under hypoxia with that following LUCAT1 overexpression. Meanwhile, LUCAT1 knockdown reversed the altered alternative splicing pattern under hypoxia (Fig. 5i, Additional file 1: Figure S14B and C). These results indicated that PTBP1 is a functional downstream effector of LUCAT1 and the splicing events regulated by LUCAT1/PTBP1 axis is also the case in CRC cells under hypoxia.

### High expression of LUCAT1 confers drug resistance to CRC cells

**Fig. 6:**
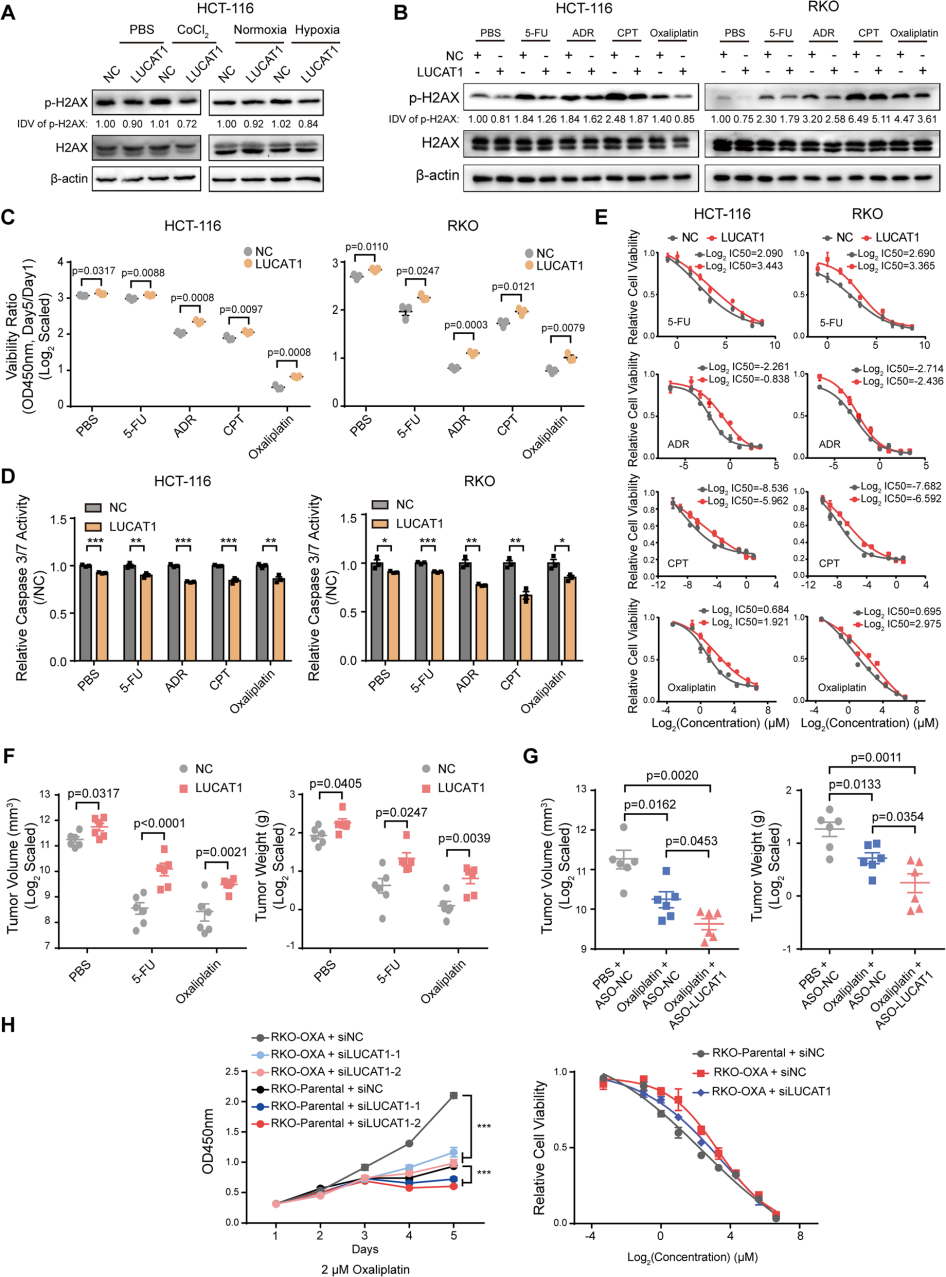
High LUCAT1 confers CRC cells to chemotherapy resistance. **a** Western blotting for p-H2AX in NC or LUCAT1 overexpressing HCT-116 cells following indicated treatment. Integrated Density Value (IDV) was obtained by ImageJ and normalized by the first lane. **b** Western blotting for p-H2AX in NC or LUCAT1 overexpressing CRC cells treated with various drugs. Integrated Density Value (IDV) was obtained by ImageJ and normalized by the first lane. **c** CCK8 assays of CRC cells treated with various drugs. Viability ratio was represented with the OD450nm ratio by day 5 to day 1. *n* = 3 independent experiments, two-tailed Student’s t-test. **d** Caspase 3/7 assays were conducted in NC or LUCAT1 overexpressing CRC cells treated with various drugs. *n* = 3 independent experiments, two-tailed Student’s t-test. **e** Viability of NC or LUCAT1 overexpressing CRC cells treated with serial indicated doses of chemotherapeutic drugs. *n* = 3 independent experiments. **f** In vivo analysis of tumor volumes and weights in mice harboring NC or LUCAT1 overexpressing HCT-116 cells treated with PBS, 5-FU and Oxaliplatin. *n* = 6 tumors, two-tailed Student’s t-test. **g** In vivo analysis of tumor volumes and weights in mice with indicated treatment. *n* = 6 tumors, two-tailed Student’s t-test. **h** CCK8 assay and viability assay of RKO-parental and RKO-OXA cells transfected with siNC or LUCAT1 siRNAs, treated with 2 μM Oxaliplatin and treated with serial indicated doses of Oxaliplatin. *n* = 3 independent experiments, two-tailed Student’s t-test. * *p* < 0.05, ** *p* < 0.01, and *** *p* < 0.001

Hypoxic tumors are refractory to chemotherapy, which is an unresolved problem in various cancers, including CRC. High expression of LUCAT1 reduced DNA damage in HCT-116 cells both under normoxia and under hypoxia or CoCl_2_ treatment, which implicated that LUCAT1 might have a role in chemoresistance of hypoxic CRC cells **(**Fig. 6a**,** Additional file 1: Figure S15A and B). Besides, chemotherapeutic drugs that are currently being adopted for the treatment of CRC, such as, 5-fluorouracil (5-FU), Camptothecin (CPT), Adriamycin (ADR) and Oxaliplatin, are DNA damage inducing reagents [22]. When treated with these chemotherapeutic drugs, CRC cells overexpressing LUCAT1 have a reduced phosphorylated H2AX compared with control cells (Fig. 6b and Additional file 1: Figure S15C). In addition, CRC cells overexpressing LUCAT1 obtained the growth advantage over the control CRC cells when treated with these DNA damage drugs (Fig. 6c). The results of caspase 3/7 activity assays also indicated that high LUCAT1 expression conferred increased resistance to DNA damage drug-induced apoptosis compared with control cells (Fig. 6d). Cells overexpressing LUCAT1 were less sensitive to these chemotherapeutic drugs treatment (Fig. 6e). Most importantly, xenograft mouse models corroborated that LUCAT1 could significantly confer resistance to 5-FU and Oxaliplatin treatment in CRC tumors in vivo (Fig. 6f and Additional file 1: Figure S15D). Next, we evaluated whether knockdown LUCAT1 has a therapeutic benefit in CRC. We treated the mice bearing CRC xenograft with intratumoral injection of LUCAT1 ASO together with Oxaliplatin. Results showed that Oxaliplatin combined with LUCAT1 ASO gets a better outcome, compared to the group treated with Oxaliplatin only, which implicates LUCAT1 could act as a therapeutic target for CRC treatment (Fig. 6g and Additional file 1: Figure S15D). Further, we established Oxaliplatin-resistant RKO cells (RKO-OXA). A higher expression of LUCAT1 was observed in RKO-OXA, compared to parental cells (Additional file 1: Figure S15E). Knockdown of LUCAT1 significantly inhibited the growth of RKO-OXA treated with Oxaliplatin, and make RKO-OXA more sensitive to Oxaliplatin (Fig. 6h). Collectively, these results demonstrate that high LUCAT1 confers drug resistance in CRC cells, which implicates LUCAT1 could act as a therapeutic target for CRC treatment.

### CRC patients with high LUCAT1 have a worse prognosis and poorly respond to chemotherapy

**Fig. 7:**
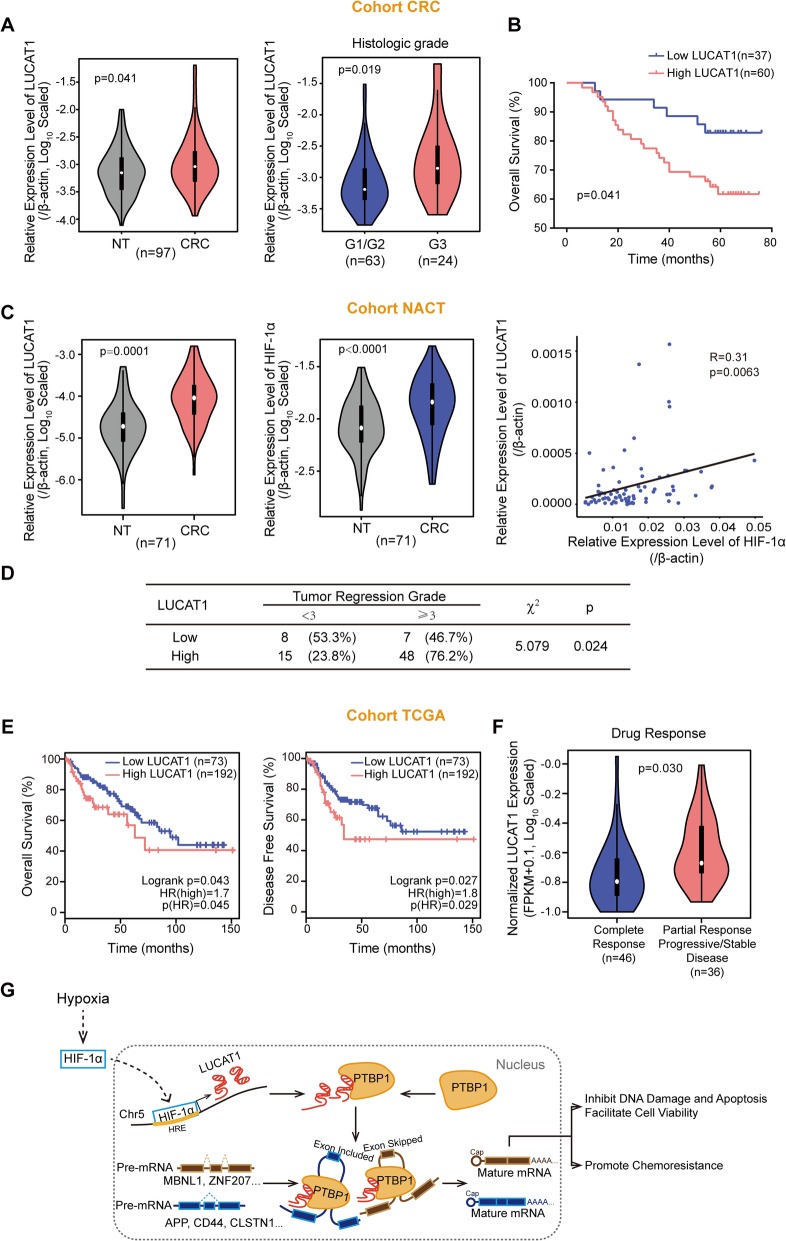
High LUCAT1 is associated with worse prognosis and poor response to chemotherapeutic drugs. **a** The expression of LUCAT1 in 97 paired CRC and adjacent normal (NT) tissues. *n* = 97 in CRC cohort, paired and unpaired Student’s t-test. **b** Kaplan–Meier analysis of overall survival curve in CRC patients with high LUCAT1 expression versus low LUCAT1 expression. *n* = 97 in CRC cohort. **c** The expression of LUCAT1 and HIF-1α in NACT cohort, and the correlation between LUCAT1 and HIF-1α in this cohort. *n* = 71 in NACT cohort, paired Student’s t-test and pearson correlation. **d** The association between tumor regression grade and HIF-1α or LUCAT1 in the NACT cohort. *n* = 78 in NACT cohort, Chi-squared test. **e** Kaplan–Meier analysis of overall survival or disease free survival curve in COAD patients with high LUCAT1 expression versus low LUCAT1 expression from TCGA cohort. Graphs were obtained from GEPIA (http://gepia.cancer-pku.cn/). **f** The expression of LUCAT1 in COAD patients with complete or partial response to drugs from TCGA cohort. *n* = 82 in TCGA-COAD, two-tailed Student’s t-test. **g** The graphic illustration demonstrating HIF-1α induced LUCAT1/PTBP1 axis in cancer

To investigate the clinical significance of LUCAT1, we analyzed 97 paired CRC tissues and found that LUCAT1 was significantly upregulated in CRC compared to adjacent tissues (Fig. 7a). CRC patients with poor histologic grades had higher expression of LUCAT1 (Fig. 7a and Additional file 1: Table S7). Those with higher expression of LUCAT1 had a worse prognosis (Fig. 7b). To determine the role of LUCAT1 in chemoresistance in patients, we collected 71 paired tissues from patients who underwent neoadjuvant chemotherapy (NACT) prior to surgery and examined the expression of LUCAT1 and HIF-1α in these tissues. Our results demonstrated that LUCAT1 and HIF-1α expression was upregulated in these CRC tissues and that the expression of LUCAT1 was positively correlated with that of HIF-1α (Fig. 7c). Notably, high expression of LUCAT1 was associated with worse tumor regression grades (TRG) (Fig. 7d). These results were validated in another TCGA cohort, which showed that patients with higher LUCAT1 expression had a worse prognosis in both overall survival and disease-free survival (Fig. 7e). Moreover, in this adjuvant chemotherapy cohort from TCGA, LUCAT1 expression is significantly higher in patients with partial or no response to chemotherapeutic drugs than that in patients with complete response to these drugs (Fig. 7f). These findings suggest that high LUCAT1 represents a subgroup of patients with poor response to chemotherapy and that LUCAT1 may act as indicator for CRC patients prior to chemotherapy.

## Discussion

In the current study, we identified that LUCAT1 is a new player for CRC cells response to hypoxia. LUCAT1 is transcribed by HIF-1α under hypoxia and regulates growth, apoptosis, and DNA damage of CRC cells. LUCAT1 overexpression confers chemotherapeutic resistance to CRC cells both in vitro and in vivo. To the best of our knowledge, this is the first report that LUCAT1 is a crucial player in hypoxia signaling pathway and chemoresistance in CRC cells. Notably, LUCAT1 is upregulated in CRC and patients with high level of LUCAT1 show a poor overall survival and disease-free survival, and respond poorly to chemotherapy. These findings suggest that LUCAT1 may act as a predictor for drug response in CRC patients and might be a useful biomarker for precise therapy in the clinic.

Previous reports demonstrated that LUCAT1 is often upregulated in several types of cancer cells and promotes the proliferation, invasion or migration of these cells from many cancer types including CRC [23–33]. LUCAT1 can act as microRNA sponge [25, 29–32], or bind proteins, such as DNMT1, to regulate their stabilities or activities [23, 24, 26, 28]. In the present study, we identified that hypoxia-induced LUCAT1, mainly located in the nucleus of CRC cells, which is physically interacts with an RNA-binding protein PTBP1 and regulates mRNA alterative splicing pathway. PTBP1 binds to several downstream transcripts, such as CD44, APP, CLSTN1, MBNL1 and ZNF207. These genes were reported to be involved in growth, DNA damage, apoptosis and drug resistance of cancer cells [34–40]. For instance, in many cancers including CRC, alternative spliced CD44 (CD44v, retained form) promotes apoptosis resistance, DNA damage resistance [36–38]. LUCAT1 enhances the binding of these downstream transcripts to PTBP1. More interestingly, the splicing events of these transcripts are correlated with LUCAT1 expression in CRC patients’ samples. These findings suggest that the alternative splicing pathway could be regulated by LUCAT1/PTBP1 interaction, and reveal a novel molecular mechanism of LUCAT1 in alternative splicing and cancer (Fig. 7g).

PTBP1 belongs to the family of heterogeneous nuclear ribonucleoproteins (hnRNPs) and regulates pre-RNA processing, especially alternative splicing. PTBP1 acts as an oncogene in many cancers through regulating alternative splicing [41–44]. In CRC, PTBP1 expression is significantly increased in cancerous tissues and is positively correlated with poor prognosis. Knockdown of PTBP1 in CRC cells led to the inhibition of cell proliferation, CD44 alternative splicing and prolonged G_2_/M phase [18], which is consistent with our findings. Intriguingly, a recent study reported that lncRNA Pnky directly binds to PTBP1 and modulates alternative splicing of key transcripts related to neural differentiation [45]. In addition, lncRNA PNCTR can bind to PTBP1 and modulate its regulation of splicing of mRNAs related to cancer cell survival [46]. Here, we found that hypoxia-induced lncRNA LUCAT1 binds to PTBP1 to alter the splicing of a subset of transcripts related to DNA damage, apoptosis, and cell cycle. The majority of common splicing events regulated by hypoxia, LUCAT1 and PTBP1 are involved in these biological processes, suggesting that hypoxia might regulate these specific alterative splicing events through the LUCAT1/PTBP1 axis. Taken together, our data indicate that PTBP1 can interact with specific lncRNAs to manipulate the alternative splicing of various target mRNAs according to the cellular context. lncRNAs may act as a determinant for the mRNA specificity spliced by PTBP1.

In summary, LUCAT1 is induced by hypoxia and confers chemoresistance to tumor cells. LUCAT1 physically interacts with PTBP1 to modulate the alternative splicing of a set of DNA damage related genes. The newly identified LUCAT1/PTBP1 axis might be a promising therapeutic target for refractory hypoxic tumors.

## Supplementary information

**Additional file 1: **Lentivirus Production and Infection. Northern Blot Analysis. Luciferase Assay. Chromatin Immunoprecipitation (ChIP) Assay. Western Blot Analysis. Cell Cycle Analysis. RNAscope Assay. Immunofluorescence. CCK8 and Colony Formation Assays. Caspase 3/7 Assay. RNA Pull Down. RNA Immunoprecipitation (RIP) Assay. MS2-GST Pull Down. **Figure S1.** Correlations with hypoxia signature genes. **Figure S2.** Characteristic features of LUCAT1. **Figure S3.** LUCAT1 promotes the proliferation and colony formation of CRC cells. **Figure S4.** LUCAT1 inhibits DNA damage and apoptosis. **Figure S5.** LUCAT1 interacts with PTBP1 and STAU1. **Figure S6.** The analysis of PTBP1 binding motif on LUCAT1. **Figure S7**. Hypoxia and LUCAT1 didn’t affect PTBP1 expression and distribution. **Figure S8**. LUCAT1 and PTBP1 regulate the alternative splicing of target genes. **Figure S9**. Target genes regulated by LUCAT1/PTBP1 axis involve in DNA damage and apoptosis. **Figure S10**. PTBP1 promotes the proliferation and colony formation of CRC cells. **Figure S11**. PTBP1 knockdown induces DNA damage in CRC cells. **Figure S12**. PTBP1 is a functional target of LUCAT1. **Figure S13**. The suppression effect of LUCAT1 knockdown on cell growth is reduced in PTBP1 knockdown cells. **Figure S14**. LUCAT1/PTBP1 axis functions under hypoxia. **Figure S15**. LUCAT1 plays an important role in chemoresistance of CRC cells. **Table S1**. Samples of human tissues. **Table S2**. Sequences of siRNAs used in this study. **Table S3**. Sequences of qPCR primers to detect RNA expression. **Table S4**. Sequences of RT-PCR primers to detect alternative splicing. **Table S5**. 25 candidate lncRNAs. **Table S6**. Mass spectrometry protein identification results for biotinylated LUCAT1 RNA pull down. **Table S7**. Correlation of the clinicopathological features with tumor LUCAT1 expression in CRC. **Table S8**. Sequences of primers used in this study. **Table S9**. Sequences of ChIP-qPCR primers to detect HREs. **Table S10**. Antibodies used in this study

## Data Availability

The authors declare that all relevant data of this study are available within the article or from the corresponding author on reasonable request.

## Abbreviations

ChIP: Chromatin immunoprecipitation
COAD: Colon adenocarcinoma
CRC: Colorectal cancer
GSEA: Gene set enrichment analysis
HIF-1α: Hypoxia inducible factor 1 alpha
HRE: Hypoxia response element
lncRNA: Long non-coding RNA
LUCAT1: Lung cancer associated transcript 1
PTBP1: Polypyrimidine tract binding protein 1
RIP: RNA immunoprecipitation
RNA-seq: RNA sequencing
STAU1: Staufen double-stranded RNA binding protein 1
TCGA: The cancer genome atlas
TRG: Tumor regression grades
