# Supplementary material for: Hypoxia induced LUCAT1/PTBP1 axis modulates cancer cell viability and chemotherapy response
Source: Mol Cancer. 2020 Jan 21;19:11. doi: 10.1186/s12943-019-1122-z (PMC6971890; doi:10.1186/s12943-019-1122-z)
Supplement: Supplementary file 1 — Additional file 1: Lentivirus Production and Infection. Northern Blot Analysis. Luciferase Assay. Chromatin Immunoprecipitation (ChIP) Assay. Western Blot Analysis. Cell Cycle Analysis. RNAscope Assay. Immunofluorescence. CCK8 and Colony Formation Assays. Caspase 3/7 Assay. RNA Pull Down. RNA Immunoprecipitation (RIP) Assay. MS2-GST Pull Down. Figure S1. Correlations with hypoxia signature genes. Figure S2. Characteristic features of LUCAT1. Figure S3. LUCAT1 promotes the proliferation and colony formation of CRC cells. Figure S4. LUCAT1 inhibits DNA damage and apoptosis. Figure S5. LUCAT1 interacts with PTBP1 and STAU1. Figure S6. The analysis of PTBP1 binding motif on LUCAT1. Figure S7. Hypoxia and LUCAT1 didn’t affect PTBP1 expression and distribution. Figure S8. LUCAT1 and PTBP1 regulate the alternative splicing of target genes. Figure S9. Target genes regulated by LUCAT1/PTBP1 axis involve in DNA damage and apoptosis. Figure S10. PTBP1 promotes the proliferation and colony formation of CRC cells. Figure S11. PTBP1 knockdown induces DNA damage in CRC cells. Figure S12. PTBP1 is a functional target of LUCAT1. Figure S13. The suppression effect of LUCAT1 knockdown on cell growth is reduced in PTBP1 knockdown cells. Figure S14. LUCAT1/PTBP1 axis functions under hypoxia. Figure S15. LUCAT1 plays an important role in chemoresistance of CRC cells. Table S1. Samples of human tissues. Table S2. Sequences of siRNAs used in this study. Table S3. Sequences of qPCR primers to detect RNA expression. Table S4. Sequences of RT-PCR primers to detect alternative splicing. Table S5. 25 candidate lncRNAs. Table S6. Mass spectrometry protein identification results for biotinylated LUCAT1 RNA pull down. Table S7. Correlation of the clinicopathological features with tumor LUCAT1 expression in CRC. Table S8. Sequences of primers used in this study. Table S9. Sequences of ChIP-qPCR primers to detect HREs. Table S10. Antibodies used in this study [file 12943_2019_1122_MOESM1_ESM.pdf]

# Supplementary Information

# Hypoxia induced LUCAT1/PTBP1 Axis Modulates Cancer Cell Viability and Chemotherapy Response

Lin Huan[1, 4], Tianan Guo[2, 4], Yangjun Wu[1], Linguo Xu[1], Shenglin Huang[1], Ye Xu[2, *], Linhui Liang[1, 3, *], Xianghuo He[1, 3, *]

1 Fudan University Shanghai Cancer Center and Institutes of Biomedical Sciences; Department of Oncology, Shanghai Medical College, Fudan University, Shanghai 200032, China

2 Department of Colorectal Surgery, Fudan University Shanghai Cancer Center; Department of Oncology, Shanghai Medical College, Fudan University, Shanghai 200032, China

3 Key Laboratory of Breast Cancer in Shanghai, Fudan University Shanghai Cancer Center, Fudan University, Shanghai 200032, China

4 These authors contributed equally to this work

[*] Correspondence: Xianghuo He, Ph.D., xhhe@fudan.edu.cn, or Linhui Liang, PhD., lianglinhui@fudan.edu.cn, Fudan University Shanghai Cancer Center and Institutes of Biomedical Sciences, Shanghai Medical College, Fudan University; or Ye Xu, MD, xuye021@163.com, Department of Colorectal Surgery, Fudan University Shanghai Cancer Center. 1207 Rm., 2# Bldg., 270 Dong An Rd., Shanghai 200032, China. Tel.: 86-21-34777577; Fax: 86-21-64172585.

# Supplementary Methods

## Lentivirus Production and Infection

The LUCAT1 fragment was cloned into the pCDH-Puro lentiviral vector, and the ORF region of PTBP1 was cloned into the pCDH-3×Flag lentiviral vector. shRNA sequences were synthesized and cloned into the LentiGuide-Puro lentiviral vector. The empty pCDH-Puro or LentiGuide-Puro vectors were used was control. The primers used in this study are listed in **Table S8**. To generate lentiviruses, the lentiviral vector, packaging plasmid (pAX2), and VSV-G envelope plasmid (pMD2.G) were cotransfected into HEK-293T cells using Lipofectamine 2000 (Invitrogen, CA, USA). The pAX2 and pMD2.G plasmids were gifts from Dr. Didier Trono (Ecole Polytechnique Fédérale de Lausanne, 1015 Lausanne, Switzerland). Supernatants were collected 48 h after transfection and used to infect the corresponding CRC cells.

## Northern Blot Analysis

The LUCAT1 probe template was amplified by PCR (Forward Primer: AATCAACACTCCACTCAGACA; Reverse Primer: gatcactaatacgactcactataggTAGTCCCAGCTACTCAGGAG). The digoxin-labeled probe was synthesized with T7 RNA polymerase using the Northern Starter Kit (Roche, IN, USA). LUCAT1 expression was detected in HCT-116 cells using the NorthernMax Kit from Ambion (Thermo Fisher

Scientific, IL, USA) according to the manufacturer's instructions.

**Luciferase Assay**

The HIF-1α binding HRE was amplified from HCT-116 cell genomic DNA and was then subcloned into the pGL3.0-basic vector. The primers were shown in **Table S8**. CRC cells were seeded into 96-well plates at a density of 5000 cells per well. After 24 h, 5 ng of pRL-TK, 100 ng of pGL3.0-basic or pGL3.0-basic-HRE, and 5 pmol of NC or HIF-1α siRNA were transfected into each well of a 96-well plate. The next day, the corresponding wells were treated overnight with 100 μM $CoCl_2$. Firefly and Renilla luciferase activities were measured using the dual-luciferase reporter assay system (Promega, WI, USA).

**Chromatin Immunoprecipitation (ChIP) Assay**

Cultured cells were crosslinked using 1% formaldehyde. Crosslinking was terminated by adding glycine to a final concentration of 0.125 M. Cells were scraped off the dish, collected into a fresh 1.5-mL tube, and resuspended in ChIP lysis buffer supplemented with proteinase inhibitor (Bimake, Shanghai, China). Chromatin was sheared into 200~1000-bp fragments by sonication at the proper conditions. IgG or HIF-1α antibody was added to Protein A/G magnetic beads (Bimake, Shanghai, China) and rotated at room temperature. After 30 min, the chromatin mixture was added to the beads and the sample was rotated at 4°C overnight. Then the tube was subjected to a magnetic field to remove the supernatant, which

contained nonspecific fragments. The beads were washed 4 times and then eluted using MinElute Spin Columns (Qiagen, Hilden, Germany). The primers used in the ChIP assay are listed in **Table S9**.

**Western Blot Analysis**

Proteins were separated by SDS-PAGE and transferred to nitrocellulose membranes (GE, CT, USA). The membranes were blocked with nonfat milk and then probed with primary antibodies overnight at 4°C. Membranes were incubated with HRP-conjugated secondary antibodies for 1 h at room temperature. Immune complexes were detected using LumiBest ECL Reagent Solution Kit (Share-Bio, Shanghai, China). Antibodies used in this study are listed in **Table S10**.

**Cell Cycle Analysis**

Cells were seeded in six-well plates, deprived of serum overnight for synchronization, and then released from synchronization by adding complete medium. After 12 h, cells were collected and fixed with 70% ethanol. Prior to flow cytometric analysis, fixed cells were treated with ribonuclease A (TaKaRa, Tokyo, Japan) for 30 min at 37°C and then stained with propidium iodide (PI) (Sangon Biotech, Shanghai, China) for 15 min at room temperature.

**RNAscope Assay**

The LUCAT1 probe (NR_103548.1) was synthesized by Advanced Cell Diagnostics (CA, USA). TSA-based fluorescent detection was conducted

according to the manufacturer's instructions. For the detection of LUCAT1 and PTBP1 co-localization, some minor modifications were made to the procedure. The proteinase digestion time was reduced to 5 min. Following the detection of HRP signal with the TSA system, the samples were probed with an anti-PTBP1 antibody and then incubated with a TRITC-conjugated secondary antibody (Proteintech, IL, USA). DAPI (Thermo Fisher Scientific, IL, USA) was used to stain cell nuclei.

**Immunofluorescence**

Cells on glass slides were fixed in 4% paraformaldehyde for 30 min at room temperature. The samples were permeabilized in 0.25% Triton X-100 for 15 min and blocked with Immunol Staining Blocking Buffer (Beyotime, Shanghai, China). The samples were probed by H2AX (Phospho-Ser139) antibody, followed by incubation with secondary anti-body conjugated with Alexa 488 (Invitrogen, Carlsbad, CA, USA). DAPI (Thermo Fisher Scientific, IL, USA) was used to stain the nucleus of the cells.

**CCK8 and Colony Formation Assays**

Cell viability was assessed using Cell Counting Kit-8 (CCK8) (Dojindo, Kumamoto, Japan). Cells were seeded into 96-well plates, and 10 μL of CCK8 solution was added to each well once a day. Samples were incubated at 37°C for 90 min, and then absorbance was detected at 450 nm. For colony formation assays, 1000 cells were seeded into each well of a six-

well plate. After two weeks, cells were fixed and stained with 0.1% crystal violet and 20% methanol, and then colonies were counted.

To investigate cell proliferation ratio following drug treatment, cells were seeded into 96-well plates. 24 hours later, 100 nM CPT (Selleck, Shanghai, China), 5 μM 5-FU (Selleck, Shanghai, China), 5 μM Oxaliplatin (Selleck, Shanghai, China), or 50 nM ADR (Sangon Biotech, Shanghai, China) was added to the culture medium.

To explore the sensitivity of cells to each drug, 8000 cells were seeded into each well of a 96-well plate. The appropriate concentration of drug was added to each well. After 48 h, cell viability was determined using the CCK8 assay.

**Caspase 3/7 Assay**

Caspase 3/7 assay was conducted using the Caspase-Glo 3/7 Assay System (Promega, WI, USA) according to the manufacturer's instructions. Briefly, 5000 cells were seeded in each well of a 96-well plate one day prior to treatment. Then, siRNAs were transfected into the cells. After 48 h, the medium was removed, and proluminescent substrates were added to each well in the dark. The luminescence of each sample was measured using a multimode reader.

To evaluate caspase 3/7 activities following drug treatments, 1 μM CPT, 100 μM 5-FU, 20 μM Oxaliplatin, or 1 μM ADR was added 24 h prior to the conduction of caspase 3/7 assay.

**RNA Pull Down**

Cells in dishes were scraped and collected in tubes by centrifugation. Cells were then lysed in RIPA buffer supplemented with RNase inhibitor (Vazyme Biotech, Jiangsu, China) and proteinase inhibitor (Bimake, Shanghai, China). Biotin-labeled sense or antisense LUCAT1 RNA and truncated LUCAT1 fragments were transcribed *in vitro* using T7 RNA Polymerase (NEB, MA, USA). Each biotin-labeled RNA was added to an RNase-free tube, heat shocked at 90°C for 2 min and mixed with Annealing Buffer for RNA Oligos (Beyotime, Shanghai, China) at room temperature for 20 min. Equal volumes of cell lysates were added to the RNA mixture and incubated at 4°C. After 4 h, the mixture was added to 30 µl of pre-washed streptavidin magnetic beads (Thermo Fisher Scientific, IL, USA) and rotated at room temperature for 1 h. The magnetic beads were washed three times with NT2 buffer and heated for 10 min at 100°C with SDS loading buffer. The protein samples were separated by SDS-PAGE and silver-stained using the Fast Silver Stain Kit (Beyotime, Shanghai, China). This sample preparation was subjected to mass spectrometry analysis or western blotting.

**RNA Immunoprecipitation (RIP) Assay**

Cells grown in dishes were crosslinked with ultraviolet light and then collected in an RNase-free tube. An equal volume of RIP lysis buffer supplemented with RNase and proteinase inhibitors was added to the tube.

The samples were stored at -80°C before use. The supernatant was collected by centrifugation for 20 min at 10,000×g at 4°C. Anti-IgG, PTBP1 or STAU1 antibodies were added to Protein A/G magnetic beads and rotated at room temperature for 30 min, respectively. Then, unlabeled antibodies were removed from the beads. Next, 100 μl of supernatant was combined with 860 μl of NT2 buffer, 35 μl of 0.5 M EDTA and 5 μl of RNase inhibitor and then added to the IgG or PTBP1-labeled beads. This mixture was rotated overnight at 4°C. The beads were washed 6 times with NT2 buffer, and then 4/5 of the beads were lysed in Trizol and 1/5 of the beads were lysed in SDS loading buffer for western blotting. The RNA was extracted with Trizol, diluted in an equal volume of nuclease-free water and reverse-transcribed into cDNA using PrimeScript RT Master Mix (TaKaRa, Tokyo, Japan). qPCR was conducted to evaluate enrichment of the target genes of interest.

**MS2-GST Pull Down**

First, we established RKO cells stably expressing the MS2-GST fusion protein. Full-length LUCAT1 was subcloned into the lenti-sgRNA (MS2) vector; this construct was called LUCAT1-MS2. LUCAT1-MS2 or the empty vector was transfected into cells expressing the MS2-GST fusion protein. The experimental procedure was similar to that of the RIP protocol with minor modifications. Briefly, 50 μl of magneGST glutathione particles (Promega, WI, USA) was added to either LUCAT1-MS2 samples

or empty vector samples and rotated at 4℃ overnight. Then, 1/5 of the beads were lysed in Trizol and 4/5 of the beads were lysed in SDS loading buffer for western blotting.

# Supplementary Figures

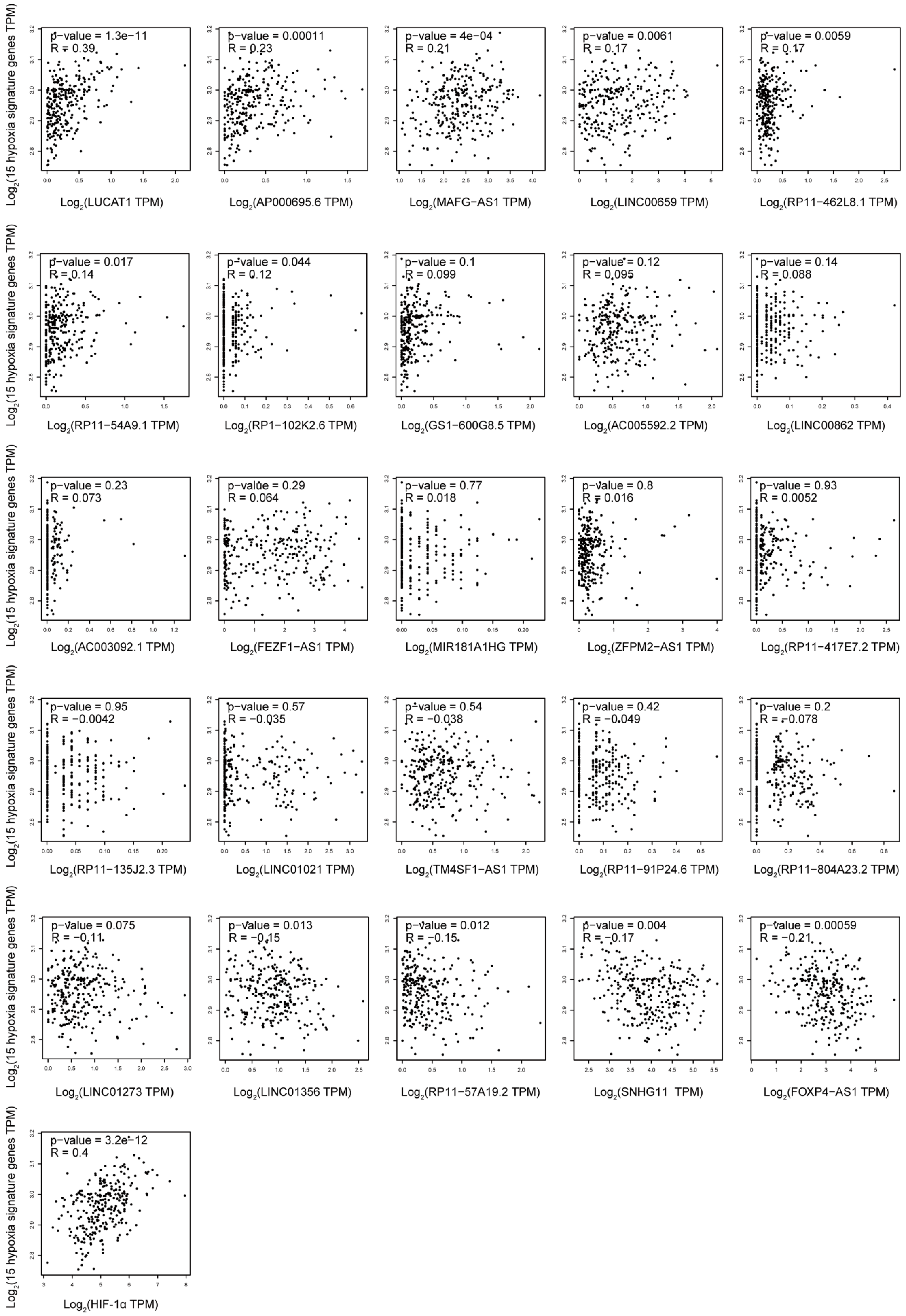


**Figure S1. Correlations with hypoxia signature genes.** Correlations between hypoxia signature genes with 25 lncRNAs were generated from GEPIA. 15 genes were chosen as hypoxia signature genes. n=275 CRC tissues in COAD. pearson correlation.

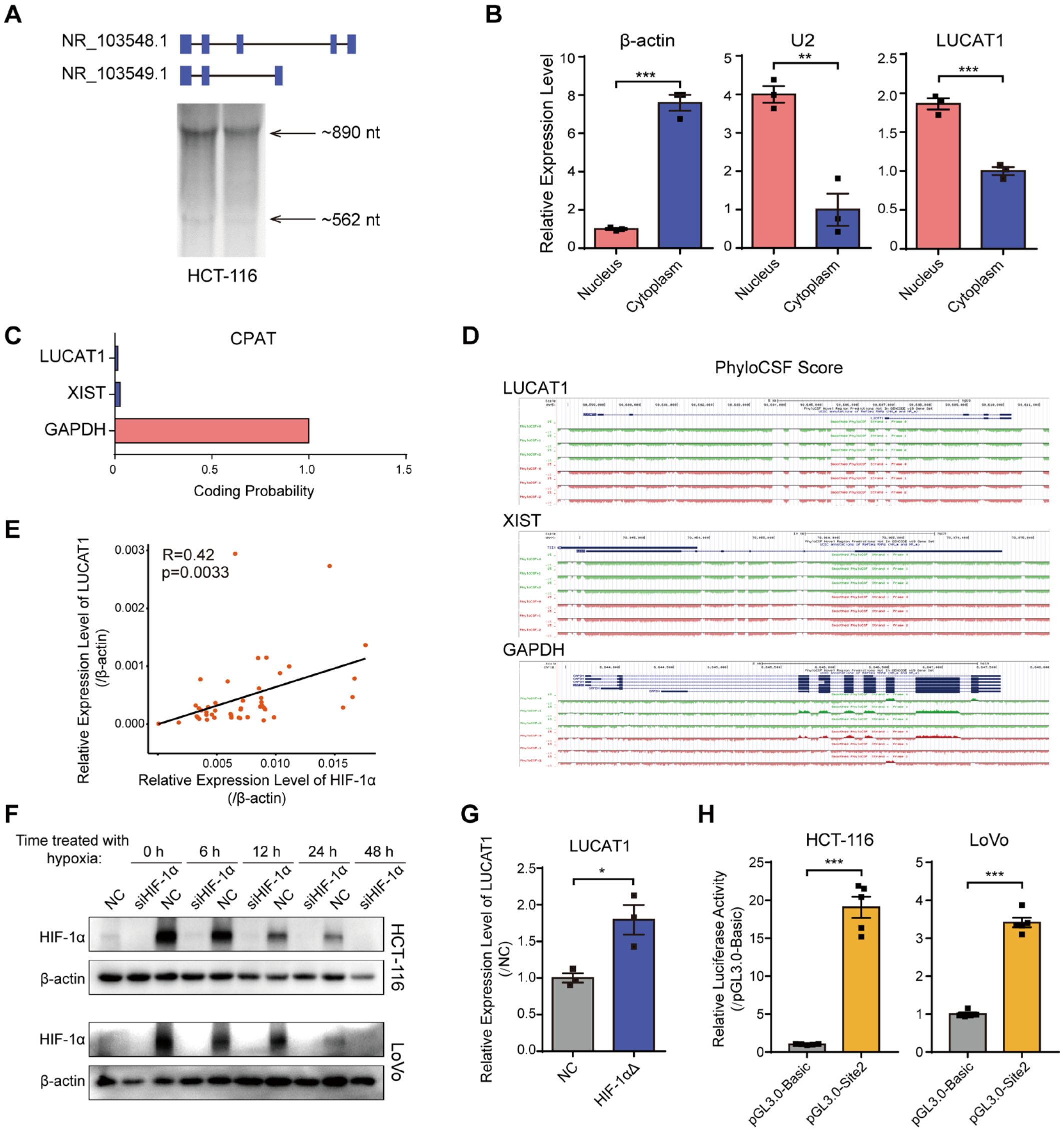


**Figure S2. Characteristic features of LUCAT1.** (A) Northern blot analysis to detect different isoforms of LUCAT1 in HCT-116 cells. (B) The distribution of LUCAT1 in HCT-116 was determined by qPCR. n=3 independent experiments, two-tailed Student's t-test. (C and D) Coding Potential Assessment Tool (CPAT, http://lilab.research.bcm.edu/cpat/) and PhyloCSF to estimate the coding probability of LUCAT1. (E) Correlation between LUCAT1 and HIF-1α in CRC tissues. n=46 in CRC cohort, pearson correlation. (F) Western blotting in HCT-116 and LoVo cells transfected with siNC or HIF-1α siRNAs under hypoxia at serial time points. (G) The expression of LUCAT1 in HCT-116 cells transfected with an empty vector (NC) or a mutant type of HIF-1α which lacks the oxygen-dependent degradation (ODD) domain. Expression level of LUCAT1 was determined by qPCR. n=3 independent experiments, two-tailed Student's t-test. (H) Transcriptional activity of the second HRE of LUCAT1 in HCT-116 and LoVo cells was determined by luciferase assay. n=5 independent experiments, two-tailed Student's t-test. * $p < 0.05$, ** $p < 0.01$, and *** $p < 0.001$.

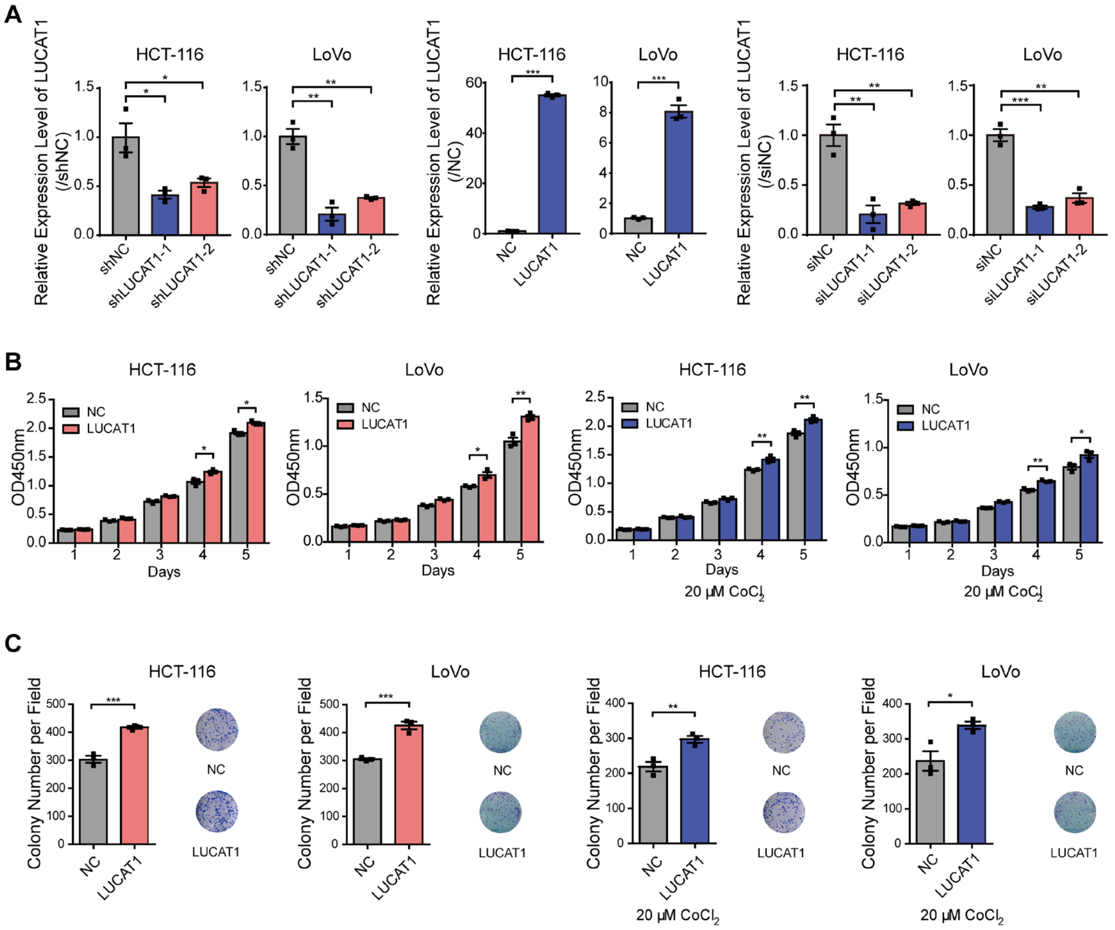


**Figure S3. LUCAT1 promotes the viability and colony formation of CRC cells.** (A) Knockdown and overexpression efficiency of LUCAT1 in CRC cells was determined by qPCR. n=3 independent experiments, two-tailed Student's t-test. (B and C) CCK8 assays and colony formations of CRC cells overexpressing NC or LUCAT1 without or with $CoCl_2$. n=3 independent experiments, two-tailed Student's t-test. * $p < 0.05$, ** $p < 0.01$, and *** $p < 0.001$.

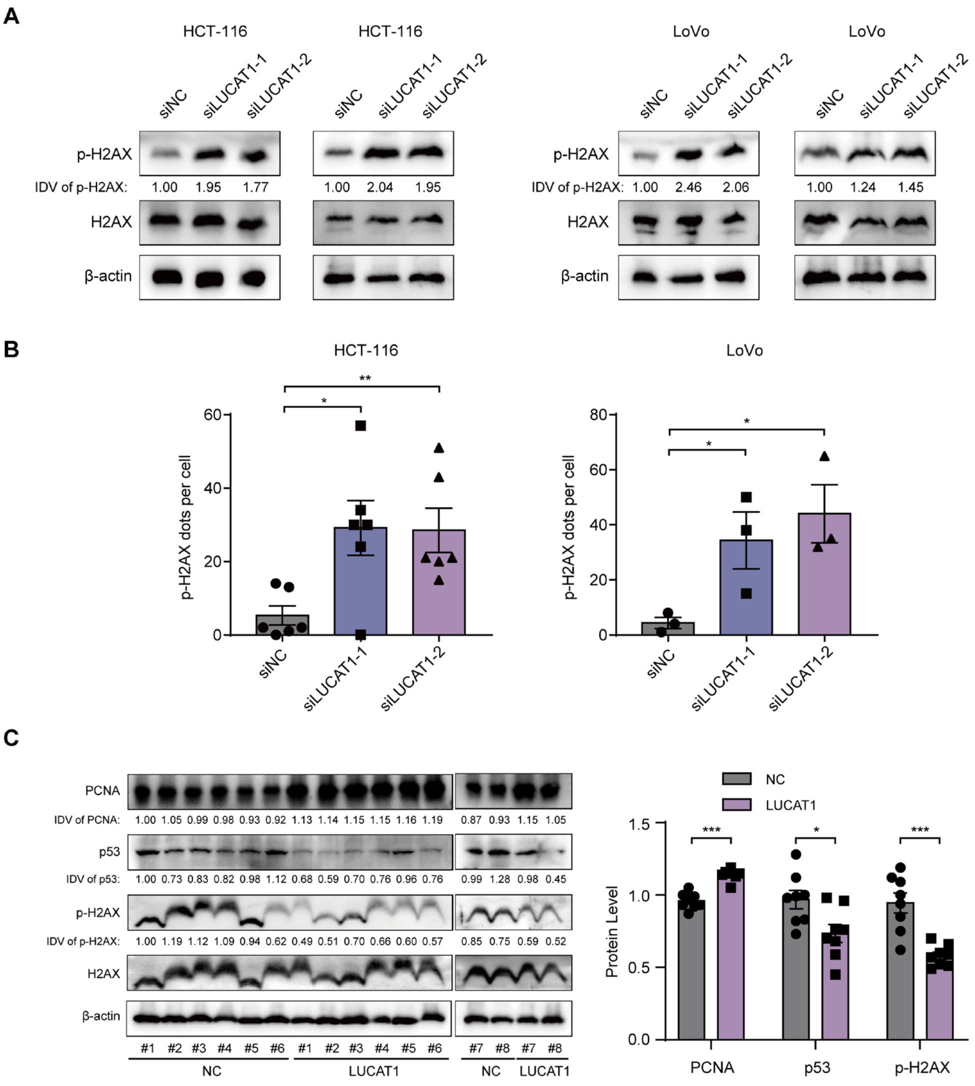


**Figure S4. LUCAT1 inhibits DNA damage and apoptosis.** (A) Replicates of p-H2AX following NC or LUCAT1 knockdown, related to Figure 2E. Integrated Density Value (IDV) was obtained by ImageJ and normalized by the first lane. (B) Dots number of p-H2AX in HCT-116 and LoVo cells after NC or LUCAT1 knockdown. n=5 or 3 independent experiments, two-tailed Student's t-test. (C) Protein expression levels of PCNA, p53 and p-H2AX in tumors. Integrated Density Value (IDV) was obtained by ImageJ and normalized by the first lane. n=8 tumors, two-tailed Student's t-test. * $p < 0.05$, ** $p < 0.01$, and *** $p < 0.001$.

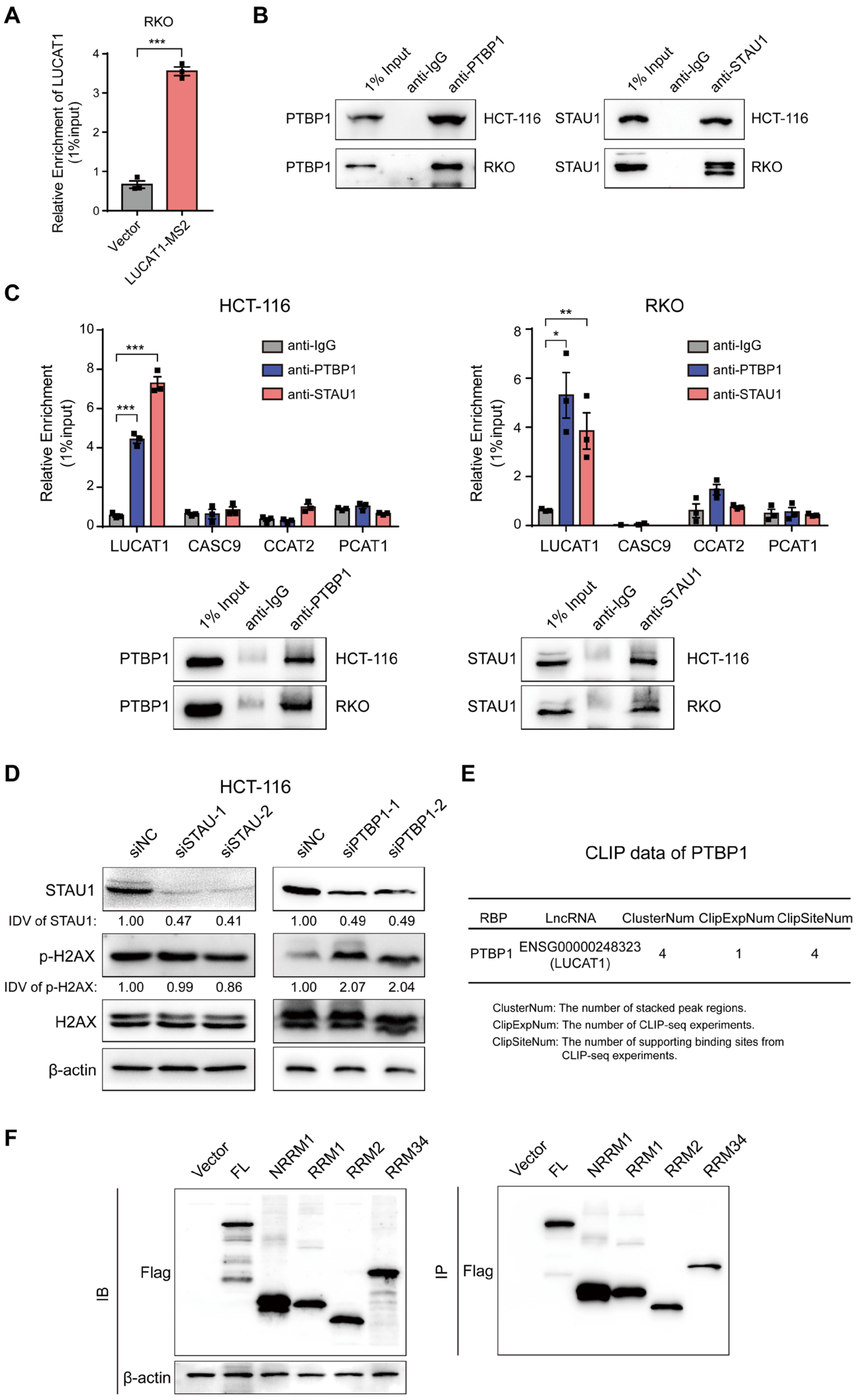


**Figure S5. LUCAT1 interacts with PTBP1 and STAU1.** (A) qPCR to detect the enrichment of LUCAT1 in MS2-based GST pull down in RKO cells. n=3 independent experiments, two-tailed Student's t-test. (B) Western blotting to detect the RIP efficiency of PTBP1 and STAU1 in CRC cells. (C) RIP assays were performed in HCT-116 and RKO cells. CASC9, CCAT2 and PCAT1 were used as control. n=3 independent experiments, two-tailed Student's t-test. (D) Western blotting in

HCT-116 transfected with siNC, PTBP1 siRNAs and STAU1 siRNAs. Integrated Density Value (IDV) was obtained by ImageJ and normalized by the first lane. (E) The CLIP data of PTBP1 from starBase (http://starbase.sysu.edu.cn/index.php). (F) Deletion mapping to identify the LUCAT1 binding domain PTBP1. Western blotting to detect the immunoprecipitation efficiency of Flag-tagged full length or truncated PTBP1 in RIP assay. * $p < 0.05$, ** $p < 0.01$, and *** $p < 0.001$.

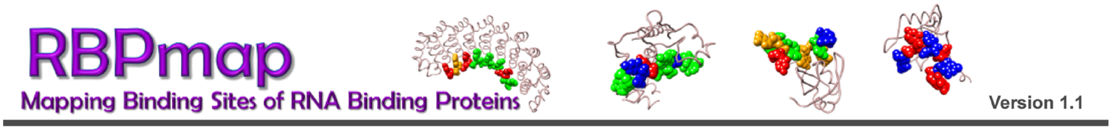


**Results for sequence:** LUCAT1

**Protein: PTBP1(Hs/Mm)**

| Position | Motif | Occurrence | Z-score | P-value |
|---|---|---|---|---|
| 88 | ucuu | cuuagacaggugcaauuuaagaacagcuuucauccucuuuucucucauauuguc | 2.161 | 1.53e-02 |
| 89 | cucucu | uuagacaggugcaauuuaagaacagcuuucauccucuuuucucucauauugucaca | 2.188 | 1.43e-02 |
| 92 | ucuu | gacaggugcaauuuaagaacagcuuucauccucuuuucucucauauugucacac | 2.161 | 1.53e-02 |
| 95 | cucucu | aggugcaauuuaagaacagcuuucauccucuuucucucauauugucacacuaugu | 2.205 | 1.37e-02 |
| 97 | cucucu | gugcaauuuaagaacagcuuucauccucuuuucucucauauugucacacuaugugu | 2.857 | 2.14e-03 |
| 98 | ucuu | ugcaauuuaagaacagcuuucauccucuuuucucucauauugucacacuaugug | 2.723 | 3.23e-03 |
| 100 | cucucu | caauuuaagaacagcuuucauccucuuuucucucauauugucacacuauguguucu | 2.205 | 1.37e-02 |
| 102 | cucucu | auuuaagaacagcuuucauccucuuuucucucauauugucacacuauguguucuga | 2.857 | 2.14e-03 |
| 103 | ucuu | uuuaagaacagcuuucauccucuuuucucucauauugucacacuauguguucug | 2.161 | 1.53e-02 |
| 104 | cucucu | uuaagaacagcuuucauccucuuuucucucauauugucacacuauguguucugacu | 2.857 | 2.14e-03 |
| 105 | ucuu | uaagaacagcuuucauccucuuuucucucauauugucacacuauguguucugac | 2.161 | 1.53e-02 |
| 107 | ucuu | agaacagcuuucauccucuuuucucucauauugucacacuauguguucugacuu | 2.161 | 1.53e-02 |
| 110 | ucuu | acagcuuucauccucuuuucucucauauugucacacuauguguucugacuucug | 2.161 | 1.53e-02 |
| 116 | cucucu | uucauccucuuuucucucauauugucacacuauguguucugacuucuggcuccuuu | 2.214 | 1.34e-02 |
| 118 | cucucu | cauccucuuuucucucauauugucacacuauguguucugacuucuggcuccuuucc | 2.214 | 1.34e-02 |
| 120 | cucucu | uccucuuuucucucauauugucacacuauguguucugacuucuggcuccuuuccuc | 2.214 | 1.34e-02 |
| 129 | cucucu | cucucauauugucacacuauguguucugacuucuggcuccuuuccucacaagaagc | 1.929 | 2.69e-02 |
| 175 | ucuu | cacaagaagcucacccagcuggaacucuuaugggaccuuggcaccagagaccac | 2.161 | 1.53e-02 |
| 185 | ucuu | ucacccagcuggaacucuuaugggaccuuggcaccagagaccacaaauuccucu | 1.884 | 2.98e-02 |
| 208 | ucuu | gaccuuggcaccagagaccacaaauuccucuuugaaguuuucuaacagcaacaa | 2.098 | 1.80e-02 |
| 210 | cucucu | ccuuggcaccagagaccacaaauuccucuuugaaguuuucuaacagcaacaauggu | 2.080 | 1.88e-02 |
| 211 | ucuu | cuuggcaccagagaccacaaauuccucuuugaaguuuucuaacagcaacaaugg | 2.580 | 4.94e-03 |
| 220 | ucuu | agagaccacaaauuccucuuugaaguuuucuaacagcaacaaugguauuucuga | 2.134 | 1.64e-02 |
| 223 | ucuu | gaccacaaauuccucuuugaaguuuucuaacagcaacaaugguauuucugacuu | 2.152 | 1.57e-02 |
| 240 | ucuu | ugaaguuuucuaacagcaacaaugguauuucugacuuggcuuucuuguauuucu | 2.161 | 1.53e-02 |
| 241 | cucucu | gaaguuuucuaacagcaacaaugguauuucugacuuggcuuucuuguauuucucuc | 2.098 | 1.80e-02 |
| 244 | ucuu | guuuucuaacagcaacaaugguauuucugacuuggcuuucuuguauuucucuca | 2.161 | 1.53e-02 |
| 245 | cucucu | uuuucuaacagcaacaaugguauuucugacuuggcuuucuuguauuucucucacgu | 2.205 | 1.37e-02 |
| 248 | ucuu | ucuaacagcaacaaugguauuucugacuuggcuuucuuguauuucucucacguu | 2.161 | 1.53e-02 |
| 253 | ucuu | cagcaacaaugguauuucugacuuggcuuucuuguauuucucucacguuaacaa | 2.161 | 1.53e-02 |
| 254 | cucucu | agcaacaaugguauuucugacuuggcuuucuuguauuucucucacguuaacaaaau | 2.848 | 2.20e-03 |
| 257 | ucuu | aacaaugguauuucugacuuggcuuucuuguauuucucucacguuaacaaaauu | 2.714 | 3.32e-03 |
| 262 | ucuu | ugguauuucugacuuggcuuucuuguauuucucucacguuaacaaaauugguuc | 2.161 | 1.53e-02 |
| 263 | cucucu | gguauuucugacuuggcuuucuuguauuucucucacguuaacaaaauugguucagc | 2.196 | 1.40e-02 |
| 265 | cucucu | uauuucugacuuggcuuucuuguauuucucucacguuaacaaaauugguucagcau | 2.848 | 2.20e-03 |
| 266 | ucuu | auuucugacuuggcuuucuuguauuucucucacguuaacaaaauugguucagca | 2.152 | 1.57e-02 |
| 267 | cucucu | uuucugacuuggcuuucuuguauuucucucacguuaacaaaauugguucagcaucu | 2.839 | 2.26e-03 |
| 268 | ucuu | uucugacuuggcuuucuuguauuucucucacguuaacaaaauugguucagcauc | 2.152 | 1.57e-02 |
| 269 | cucucu | ucugacuuggcuuucuuguauuucucucacguuaacaaaauugguucagcaucuac | 2.188 | 1.43e-02 |
| 327 | cucucu | ugggcuacaugcugagcuacagaguuucgcucugucgcccaggcuggagugcagug | 2.018 | 2.18e-02 |
| 329 | cucucu | ggcuacaugcugagcuacagaguuucgcucugucgcccaggcuggagugcaguggc | 2.580 | 4.94e-03 |
| 331 | cucucu | cuacaugcugagcuacagaguuucgcucugucgcccaggcuggagugcaguggcgc | 2.580 | 4.94e-03 |
| 333 | cucucu | acaugcugagcuacagaguuucgcucugucgcccaggcuggagugcaguggcgcgc | 2.018 | 2.18e-02 |
| 544 | ucuu | cuaaaggggcugaaugugacugacgucuuuggaaggaugagacuuagcgugccu | 2.161 | 1.53e-02 |
| 690 | ucuu | gcuacacuuaccagcugucccucaguguucuacuucuuaaaaaaagagagaugg | 2.098 | 1.80e-02 |
| 693 | ucuu | acacuuaccagcugucccucaguguucuacuucuuaaaaaaagagagauggaua | 2.098 | 1.80e-02 |
| 696 | ucuu | cuuaccagcugucccucaguguucuacuucuuaaaaaaagagagauggauaaac | 2.027 | 2.13e-02 |
| 699 | ucuu | accagcugucccucaguguucuacuucuuaaaaaaagagagauggauaaacaga | 2.580 | 4.94e-03 |
| 827 | ucuu | uucucauccccucacaaauaaagagccuucaagcucuugcagucaacaagaacu | 2.027 | 2.13e-02 |
| 836 | ucuu | ccucacaaauaaagagccuucaagcucuugcagucaacaagaacuuuuggaaug | 2.580 | 4.94e-03 |
| 853 | ucuu | cuucaagcucuugcagucaacaagaacuuuuggaaugauuucacugccugaaaa | 1.884 | 2.98e-02 |
| 855 | ucuu | ucaagcucuugcagucaacaagaacuuuuggaaugauuucacugccugaaaagg | 1.884 | 2.98e-02 |

**Figure 6. The analysis of PTBP1 binding motif on LUCAT1.** PTBP1 binding sites on LUCAT1 was predicted by RBPmap.

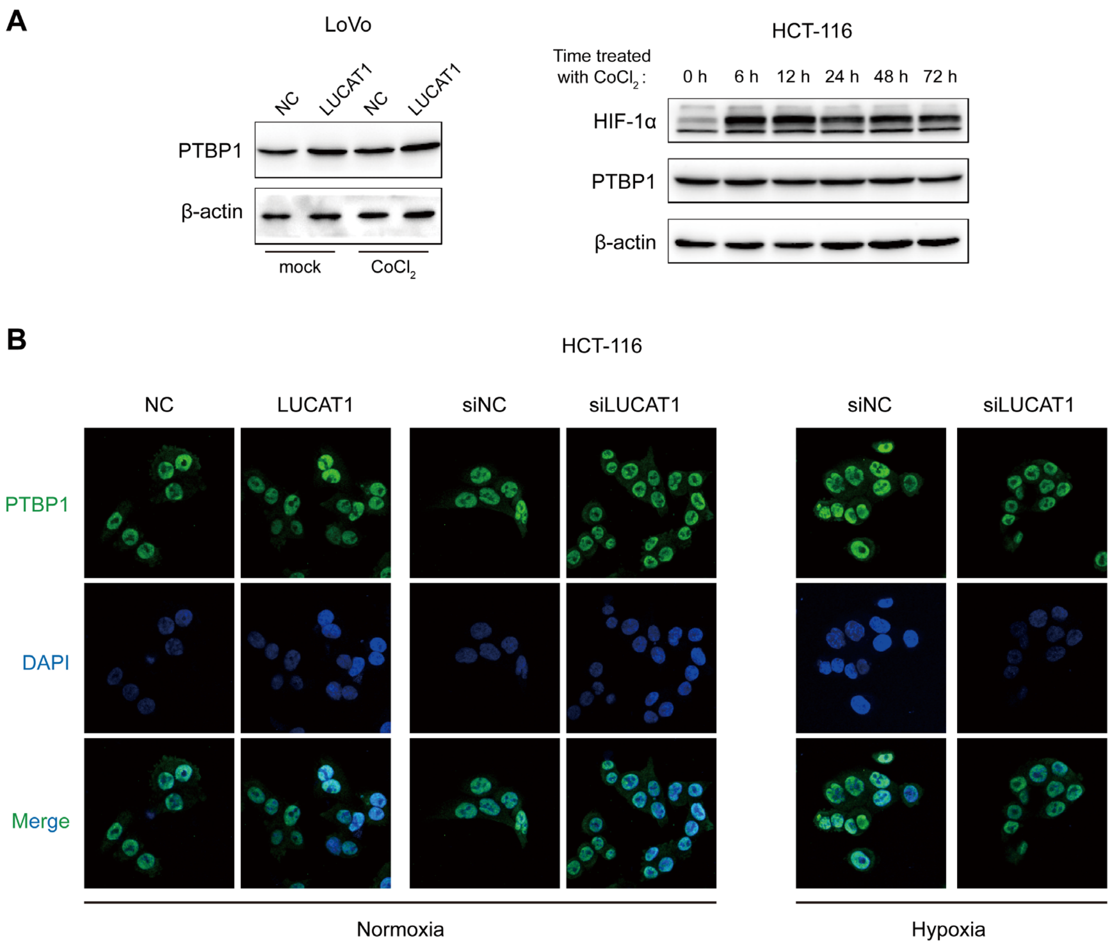


**Figure S7. Hypoxia and LUCAT1 didn't affect PTBP1 expression and distribution.** (A) Western blotting in CRC cells overexpressing NC or LUCAT1 and CRC cells treated with hypoxia at serial time points. (B) Confocal microscopic images of PTBP1 in HCT-116 cells overexpressing NC or LUCAT1 and in HCT-116 cells transfected with siNC or LUCAT1 siRNAs without or with hypoxia treatment.

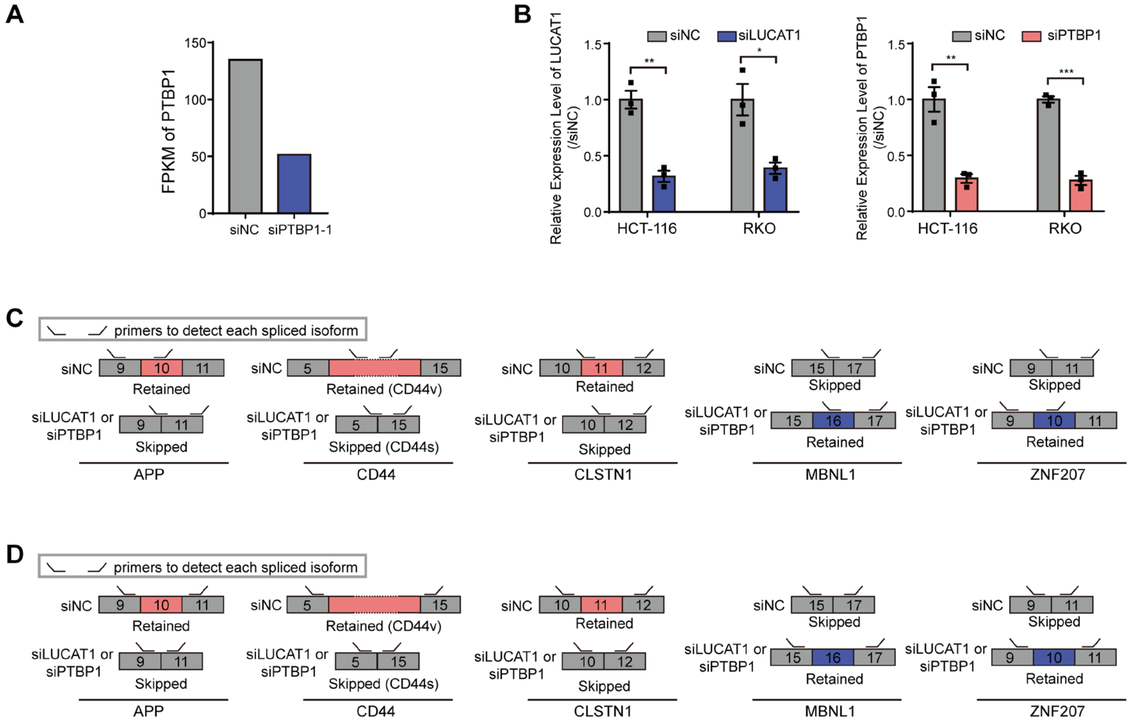


**Figure S8. LUCAT1 and PTBP1 regulate the alternative splicing of target genes.** (A) FPKM of PTBP1 in RNA-seq samples. (B) Knockdown efficiency of LUCAT1 or PTBP1 in HCT-116 and RKO was determined by qPCR. n=3 independent experiments, two-tailed Student's t-test. (C) Schematic diagrams of qPCR primers designed to detect each spliced isoform of target genes. (D) Schematic diagrams of RT-PCR primers designed to detect each spliced isoform of target genes. * $p < 0.05$, ** $p < 0.01$, and *** $p < 0.001$.

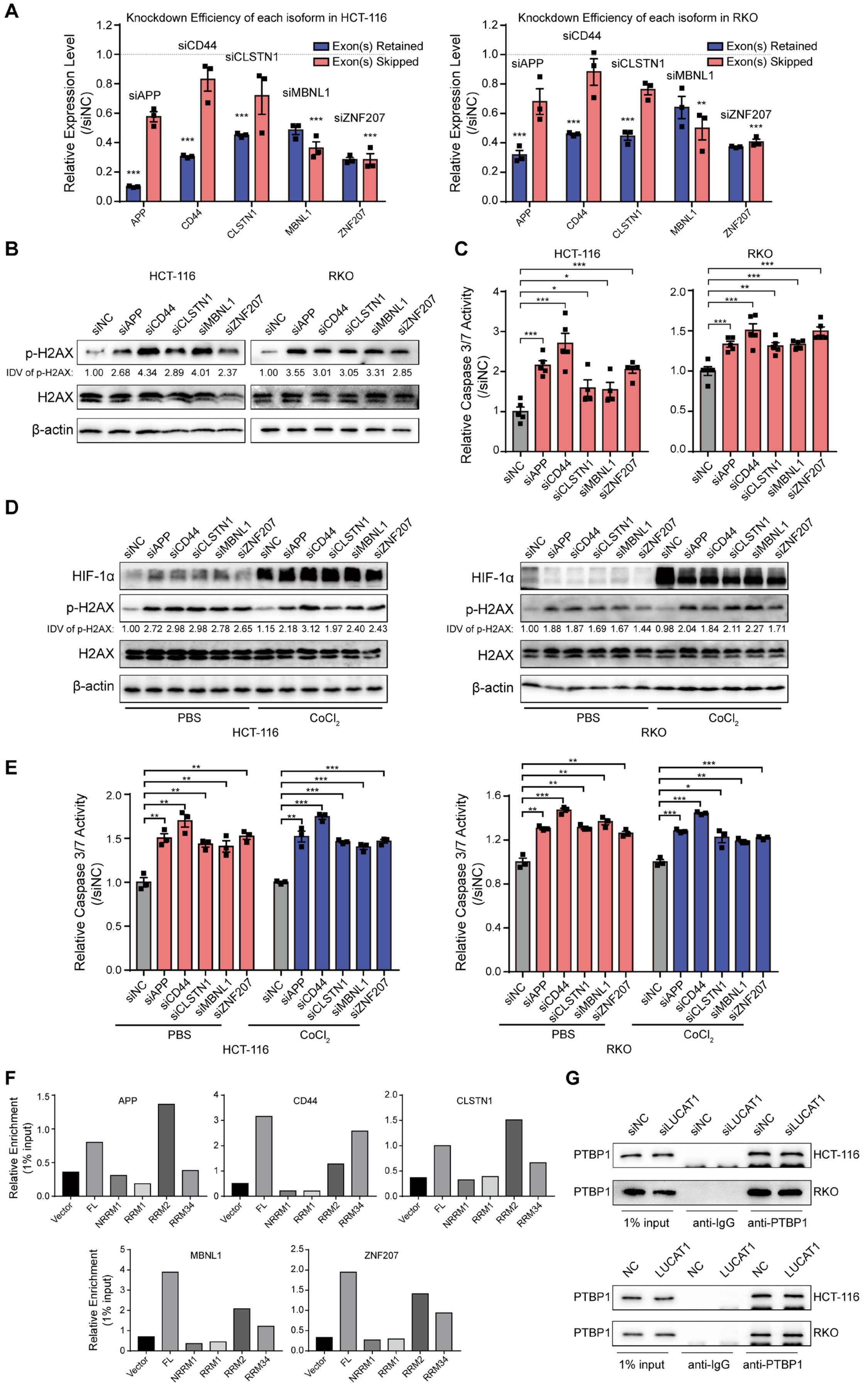
A
Knockdown Efficiency of each isoform in HCT-116
Knockdown Efficiency of each isoform in RKO
Relative Expression Level (/siNC)
Exon(s) Retained
Exon(s) Skipped
siAPP
siCD44
siCLSTN1
siMBNL1
siZNF207
APP
CD44
CLSTN1
MBNL1
ZNF207
B
HCT-116
RKO
p-H2AX
IDV of p-H2AX: 1.00 2.68 4.34 2.89 4.01 2.37
1.00 3.55 3.01 3.05 3.31 2.85
H2AX
β-actin
C
Relative Caspase 3/7 Activity (/siNC)
siNC
D
HIF-1α
IDV of p-H2AX: 1.00 2.72 2.98 2.98 2.78 2.65 1.15 2.18 3.12 1.97 2.40 2.43
IDV of p-H2AX: 1.00 1.88 1.87 1.69 1.67 1.44 0.98 2.04 1.84 2.11 2.27 1.71
PBS
CoCl2
E
F
Relative Enrichment (1% input)
Vector
FL
NRRM1
RRM1
RRM2
RRM34
G
PTBP1
siLUCAT1
NC
LUCAT1
1% input
anti-IgG
anti-PTBP1

**Figure S9. Target genes regulated by LUCAT1/PTBP1 axis involve in DNA damage and apoptosis.** (A) The expression of each isoform of target genes in CRC cells transfected with indicated siRNAs was determined by qPCR and normalized by CRC cells transfected with siNC. n=3 independent experiments, two-tailed Student's t-test. (B and C) Western blotting for p-H2AX and Caspase 3/7 activity assay of CRC cells transfected with indicated siRNAs. Integrated Density Value (IDV) was obtained by ImageJ and normalized by the first lane. n=5 independent experiments, two-tailed Student's t-test. (D) Western blotting for p-H2AX in CRC cells transfected with indicated siRNAs, without or with $CoCl_2$ treatment. Integrated Density Value (IDV) was obtained by ImageJ and normalized by the first lane. (E) Caspase 3/7 activity assay of CRC cells transfected with indicated siRNAs, without or with $CoCl_2$. n=3 independent experiments, two-tailed Student's t-test. (F) PTBP1-RIP to detect the enrichment of target genes. (G) Western blotting to detect the immunoprecipitation efficiency of PTBP1 in CRC cells. * $p < 0.05$, ** $p < 0.01$, and *** $p < 0.001$.

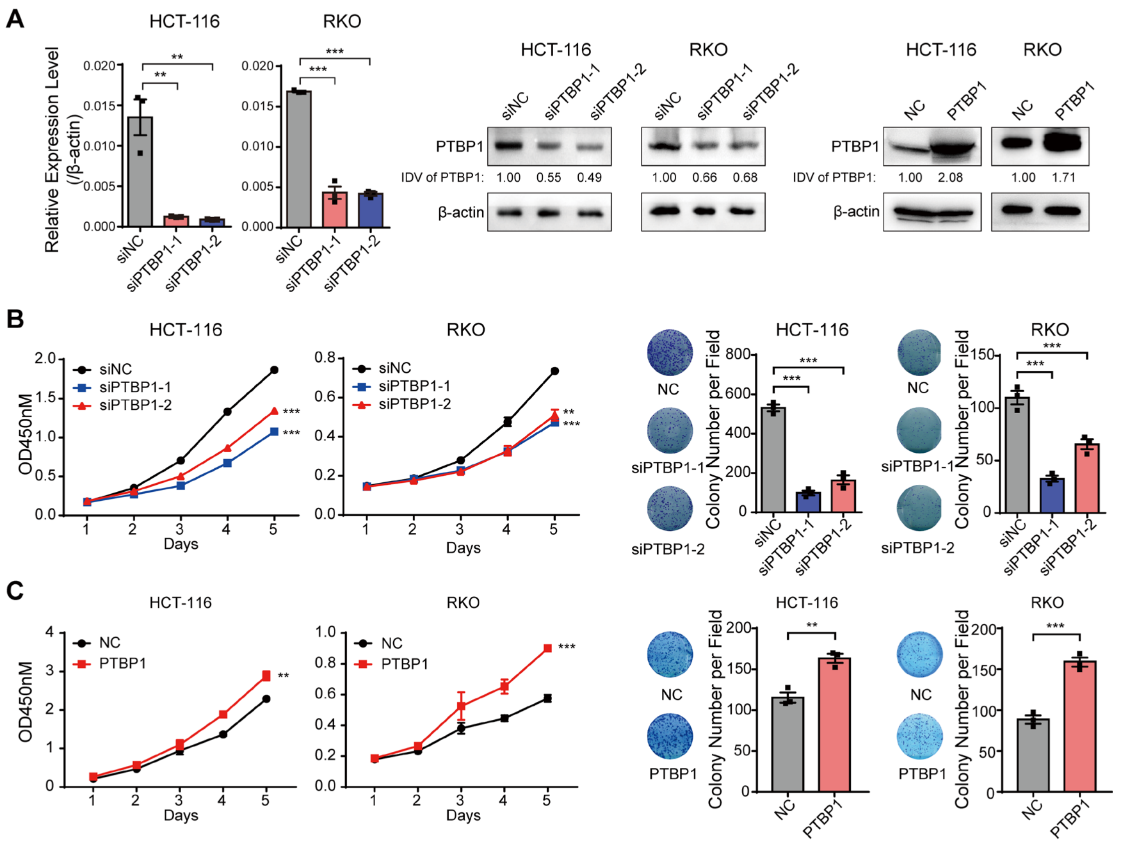


**Figure S10. PTBP1 promotes the growth and colony formation of CRC cells.** (A) Knockdown and overexpression efficiency of PTBP1 in CRC cells were determined by qPCR or western blotting. n=3 independent experiments, two-tailed Student's t-test. Integrated Density Value (IDV) was obtained by ImageJ and normalized by the first lane. (B) CCK8 assays and colony formation assays of CRC cells transfected with siNC or PTBP1 siRNAs. n=3 independent experiments, two-tailed Student's t-test. (C) CCK8 assays and colony formation assays of CRC cells overexpressing NC or PTBP1. n=3 independent experiments, two-tailed Student's t-test. * $p < 0.05$, ** $p < 0.01$, and *** $p < 0.001$.

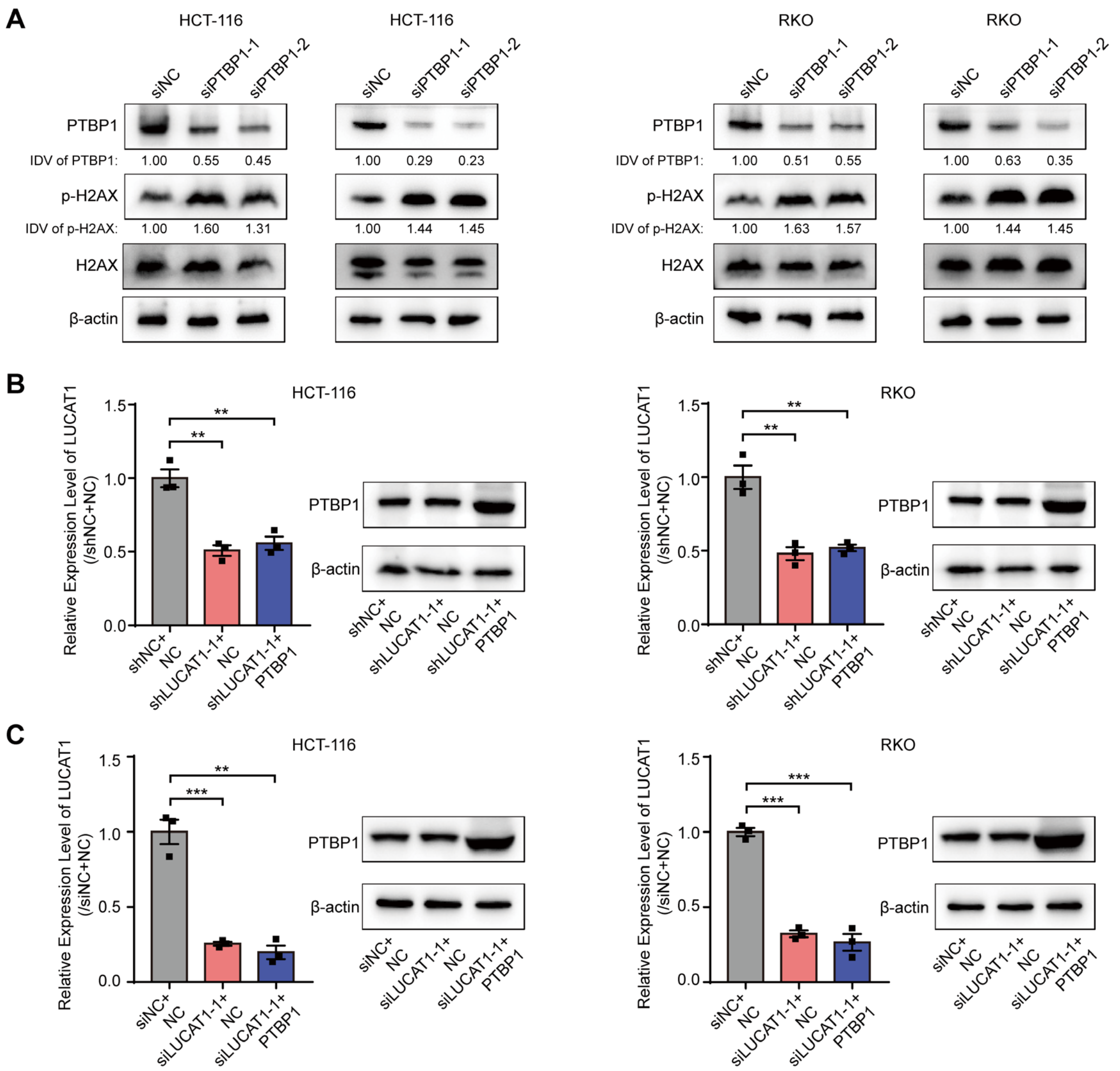


**Figure S11. PTBP1 knockdown induces DNA damage in CRC cells.** (A) Replicates of p-H2AX in CRC cells transfected with siNC or PTBP1 siRNAs, related to Figure 5B. Integrated Density Value (IDV) was obtained by ImageJ and normalized by the first lane. (B and C) Rescue efficiencies were determined by qPCR and western blotting. n=3 independent experiments, two-tailed Student's t-test. * $p < 0.05$, ** $p < 0.01$, and *** $p < 0.001$.

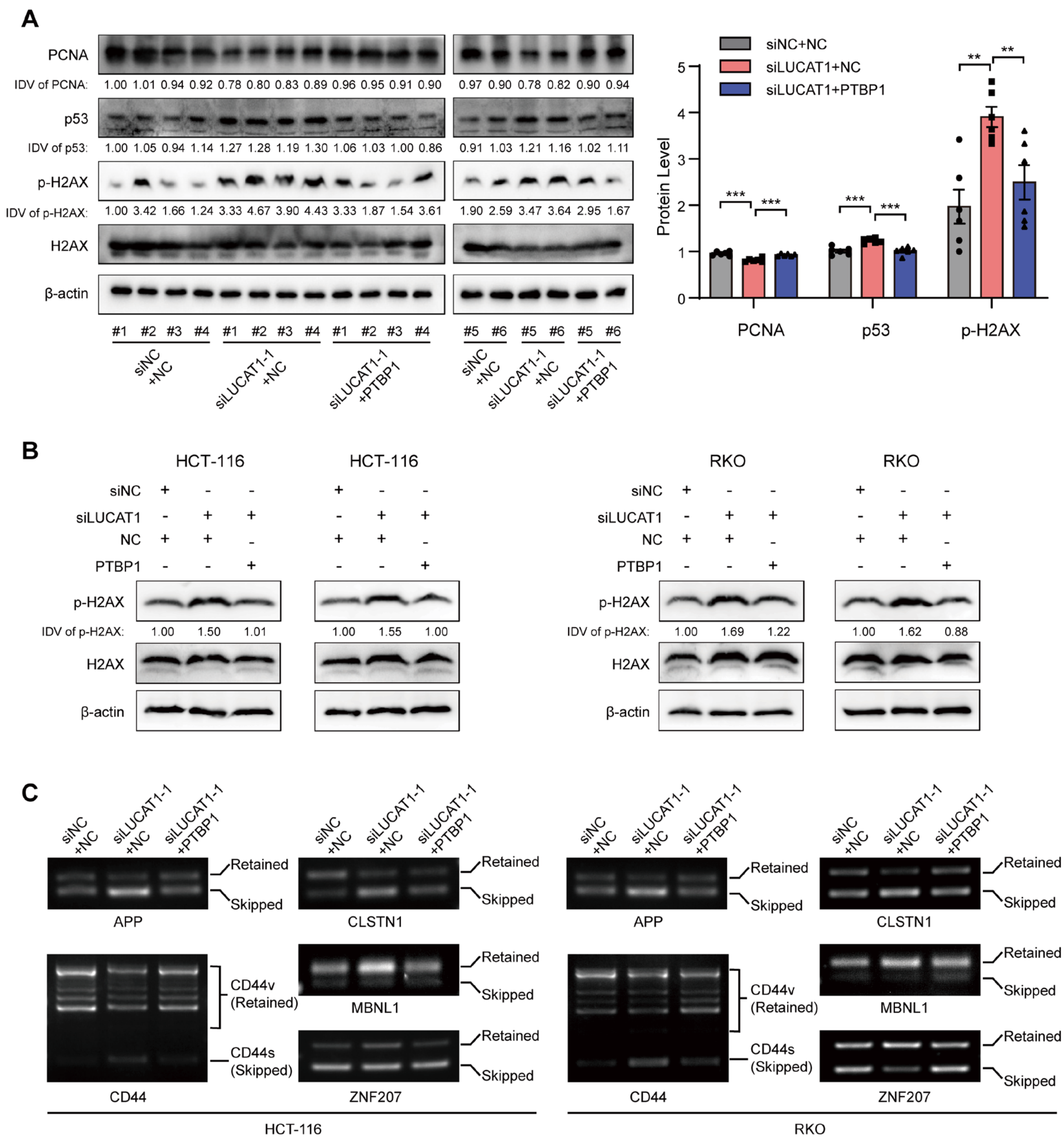


**Figure S12. PTBP1 is a functional target of LUCAT1.** (A) Protein expression levels of PCNA, p53 and p-H2AX in tumors. Integrated Density Value (IDV) was obtained by ImageJ and normalized by the first lane. n=6 tumors, two-tailed Student's t-test. (B) Replicates of western blotting for p-H2AX expression in LUCAT1-depleted CRC cells following reintroduction of PTBP1, related to Figure 5F. Integrated Density Value (IDV) was obtained by ImageJ and normalized by the first lane. (C) Alternative splicing of target genes in LUCAT1-depleted CRC cells following reintroduction of PTBP1 was validated by RT-PCR. * $p < 0.05$, ** $p < 0.01$, and *** $p < 0.001$.

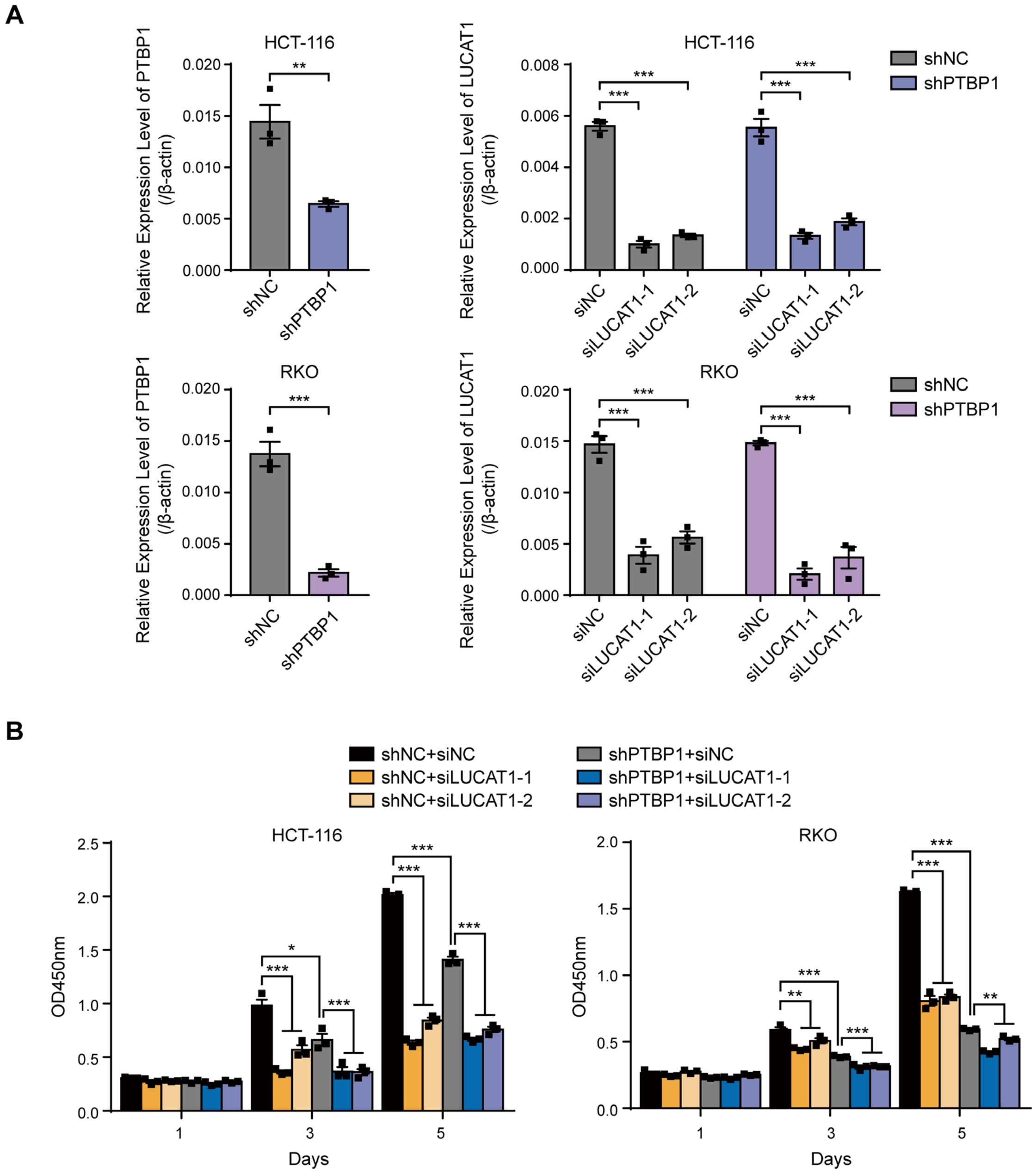


**Figure S13. The suppression effect of LUCAT1 knockdown on cell growth is reduced in PTBP1 knockdown cells.** (A) Knockdown efficiency of PTBP1 or LUCAT1 was determined by qPCR. n=3 independent experiments, two-tailed Student's t-test. (B) CCK8 assays of CRC cells with indicated treatment. n=3 independent experiments, two-tailed Student's t-test. * $p < 0.05$, ** $p < 0.01$, and *** $p < 0.001$.

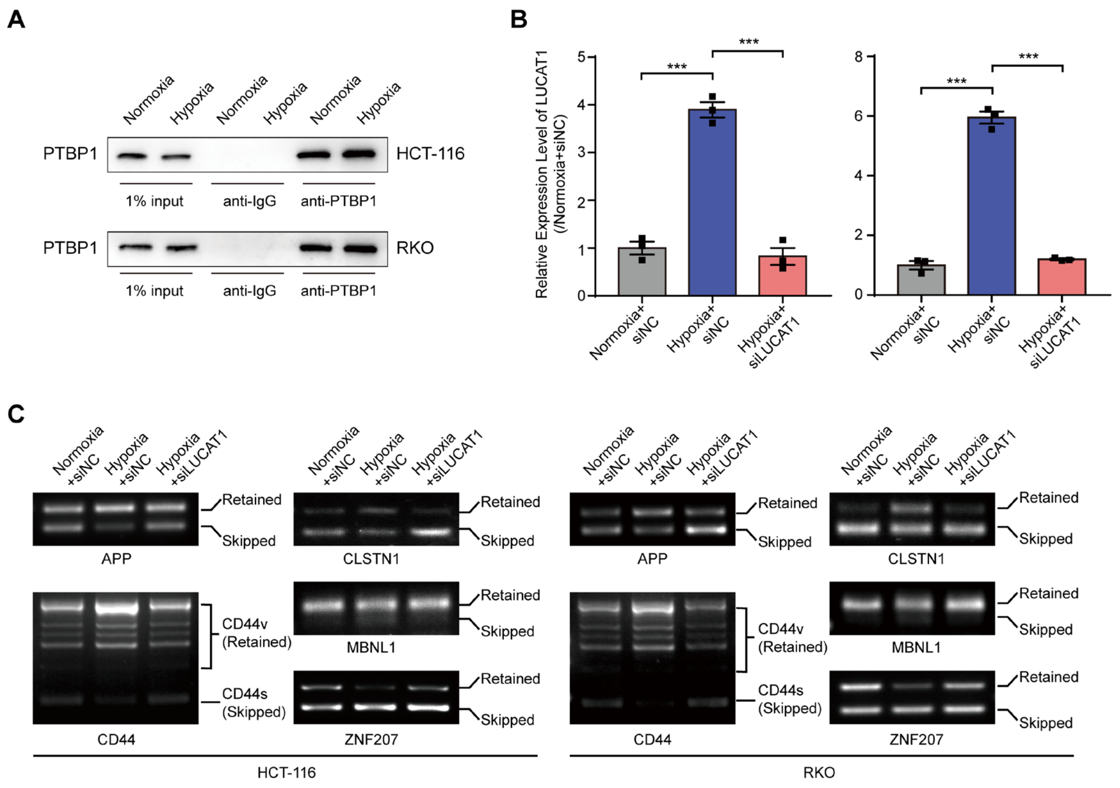


**Figure S14. LUCAT1/PTBP1 axis functions under hypoxia.** (A) Western blotting to detect the immunoprecipitation efficiency of PTBP1 in CRC cells. (B) Expression level of LUCAT1 was determined by qPCR. n=3 independent experiments, two-tailed Student's t-test. (C) Alternative splicing of target genes in CRC cells with indicated treatment was validated by RT-PCR. * $p < 0.05$, ** $p < 0.01$, and *** $p < 0.001$.

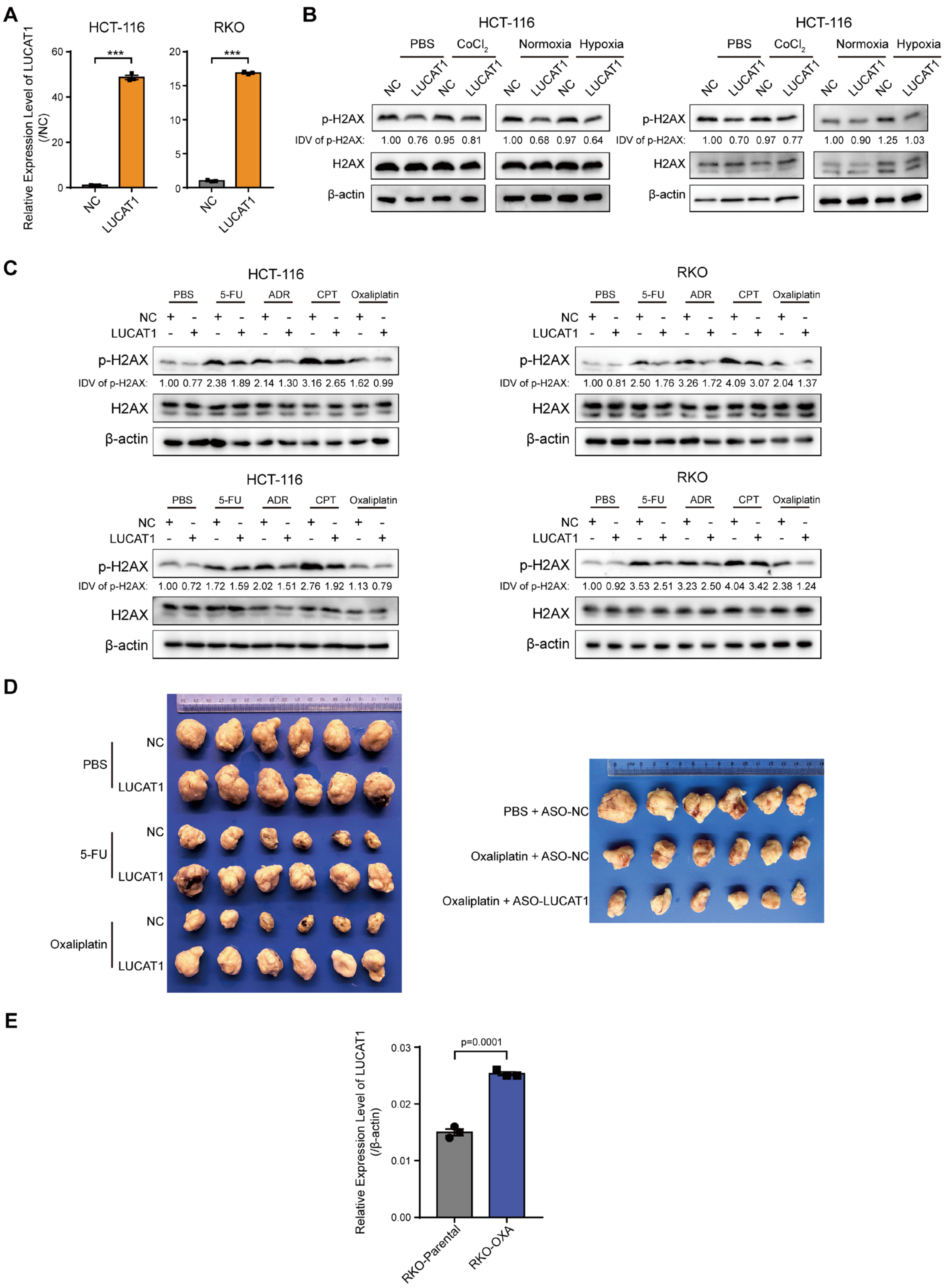


**Figure S15. LUCAT1 plays an important role in chemoresistance of CRC cells.** (A) Overexpressing efficiency of LUCAT1 in CRC cells was determined by qPCR. n=3 independent experiments, two-tailed Student's t-test. (B) Replicates of p-H2AX in in NC or LUCAT1 overexpressing HCT-116 cells following indicated treatment, related to Figure 6A. Integrated Density Value (IDV) was obtained by ImageJ and normalized by the first lane. (C) Replicates of western blotting for p-H2AX in NC or LUCAT1 overexpressing CRC cells treated with various

drugs, related to Figure 6B. Integrated Density Value (IDV) was obtained by ImageJ and normalized by the first lane. (D) Tumors of xenograft mouse model treated with indicated chemotherapeutic drugs or ASO. (E) The expression level of LUCAT1 in RKO-parental and RKO-OXA was determined by qPCR. n=3 independent experiments, two-tailed Student's t-test. * $p < 0.05$, ** $p < 0.01$, and *** $p < 0.001$.

# Supplementary Tables

## Table S1. Samples of human tissues

| Identifier | CRC tissues (No.) | Adjacent tissues (No.) | Related to |
|---|---|---|---|
| Cohort 1 | 4 | 4 | RNA-seq in Figure 1A |
| Cohort 2 | 46 | NA | Figure S2E, Figure 4H |
| Cohort CRC | 97 | 97 | Figure 7A and 7B |
| Cohort NACT | 78 | 71 | Figure 7C and 7D |

# Table S2. Sequences of siRNAs used in this study

| Identifier | Sequences (5'-3') |
|---|---|
| siHIF-1α-1 | GAAGGAACCUGAUGCUUUA |
| siHIF-1α-2 | CUGAUGACCAGCAACUUGA |
| siHIF-1α-3 | CAAUCAAGAAGUUGCAUUA |
| siLUCAT1-1 | GCUCCUUUCCUCACAAGAA |
| siLUCAT1-2 | CACACUAUGUGUUCUGACU |
| siPTBP1-1 | CAAAGCCUCUUUAUUCUUU |
| siPTBP1-2 | CUUCCAUCAUUCCAGAGAA |
| siSTAU1-1 | GCUGCCUGCUGGAAUUCUU |
| siSTAU1-2 | GCCUGCAGUUGAACGAGUA |
| siAPP | CCAAAGUUUACUCAAGACU |
| siBIN1 | GCAGAUCCUCAGCCUGUUU |
| siCD44 | GCAACUCCUAGUAGUACAA |
| siCOA1 | GCAGCUGUUGUCUUCCAAC |
| siCLSTN1 | GACAAUGAAACUGAGCCUG |
| siZNF207 | UGUUGAAGCUGUAGACUGU |
| siMBNL1 | GCGGGCAUCUUGGAAUCAU |

# Table S3. Sequences of qPCR primers to detect RNA expression

| Identifier | Forward Primer | Reverse Primer |
|---|---|---|
| β-actin | TTGTTACAGGAAGTCCCTTGCC | ATGCTATCACCTCCCCTGTGTG |
| LUCAT1 | TTGGCACCAGAGACCACAAA | GGGCGACAGAGCGAAACTCT |
| APP | TCAGGGACCAAAACCTGCAT | CTCACCAACTAAGCAGCGGT |
| CD44 | AAGTTTTGGTGGCACGCAGC | TCCACGTGGAATACACCTGC |
| CLSTN1 | CTGGACTGTGAGCTGCAGAA | TGACCGTGGCTTTGTAGGAC |
| MBNL1 | CCCAGCAAATGCAACTAGCC | ACTGAAAACATTGGCACGGG |
| ZNF207 | GCCGTGGTGCTGGTATTGTA | TGCTTTGCTTTTTGGTGCTGA |
| APP_Exon10_Retained | CAGCGCCATGTCCCAAAGTT | TGGCCTCAAGCCTCTCTTTG |
| APP_Exon10_Skipped | GGCAGCGCCATTCCTACAA | TTGACGTTCTGCCTCTTCCC |
| CD44v_Exons_Retained | ATCGCCAAACACCCAAAGAA | CTTCCTTGCATTGGATGGCT |
| CD44s_Exons_Skipped | AAAGGAGCAGCACTTCAGGA | TGTGTCTTGGTCTCTGGTAGC |
| CLSTN1_Exon11_Retained | TGCTGGCAAGAGTTTTCAGGA | TCTTATCCGCGAGTTTCCCG |
| CLSTN1_Exon11_Skipped | GCTGGCAAGGTGGCGA | TCTTATCCGCGAGTTTCCCG |
| MBNL1_Exon16_Retained | CATTTCTCCCACCAGGCTCA | GTATCTGGTTGGCTGTGGCT |
| MBNL1_Exon16_Skipped | ACAGCATTTCTCCCACCAGT | TGGGTATCTGGTTGGCTGTG |
| ZNF207_Exon10_Retained | TTCCCCAGTGCTGGACAGAT | CCTGAGCTTGTGCTGTGCTA |
| ZNF207_Exon10_Skipped | CCAGTGCTGGACAGGCTC | GGAATGTAGGCTTTGGGGGT |
| CASC9 | AGATGAAGCCGGTACCTCAGAT | TCACTTTAAAGAGGGAGAGGAG |
| CCAT2 | TGAAGGCGTCGTCCAAATGA | GGGCTGTGGAAGTGGAATCA |
| PCAT1 | GAGAGCTGACATAGGCACCC | TCTCCACTGGTGTTCATGGC |

# Table S4. Sequences of RT-PCR primers to detect alternative splicing

| Identifier | Forward Primer | Reverse Primer |
|---|---|---|
| APP | CCCATTCTTTTACGGCGGAT | CCTCTCTTTGGCTTTCTGGA |
| CD44 | TTCAGGAGGTTACATCTTTT | TAGGACCAGAGGTTGTGTTT |
| CLSTN1 | TATGTGGATGGCACGTCCCA | TTAAGCCAGCCAGATTGCCT |
| MBNL1 | TAGCCAACATGCAGTTACAA | TTGTGGCAGATGTTGTTGCT |
| ZNF207 | AGCACAGGCTGTTTCAGCGC | GGCTTTGGGGGTTCTGTAGTT |

# Table S5. 25 candidate lncRNAs

| GENEID | FC in hypoxia | $log_2$FC in COAD | FPKM in RKO cell | correlation with HIF-1α | p value of correlation |
|---|---|---|---|---|---|
| LUCAT1 | 8.8713877 | 3.1746281 | 1.992026 | 0.4424882 | 7.29E-26 |
| MIR181A1HG | 4.2892538 | 3.1002357 | 0.324943 | 0.3953506 | 1.58E-20 |
| LINC00862 | 2.76684 | 2.5980567 | 0.149837 | 0.374900661 | 1.83E-18 |
| AP000695.6 | 2.2576551 | 1.8896289 | 0.220147 | 0.361367019 | 3.54E-17 |
| RP11-135J2.3 | 2.197264 | 2.1745429 | 0.759343 | 0.27930647 | 1.36E-10 |
| RP11-417E7.2 | 3.2861207 | 3.7051105 | 0.104746 | 0.2350017 | 7.90E-08 |
| ZFPM2-AS1 | 3.2024376 | 2.1506945 | 0.158024 | 0.2185239 | 6.25E-07 |
| RP1-102K2.6 | 2.0546735 | 2.3303453 | 0.151847 | 0.202881137 | 3.86382E-06 |
| GS1-600G8.5 | 2.0307802 | 2.5661858 | 0.117933 | 0.189112681 | 1.71462E-05 |
| AC003092.1 | 9.9928214 | 1.1410818 | 0.377648 | 0.1713429 | 0.000100754 |
| RP11-54A9.1 | 2.7675406 | 2.2201079 | 0.314984 | 0.141992113 | 0.00130413 |
| FEZF1-AS1 | 4.2416514 | 8.8615013 | 0.106695 | 0.1090304 | 0.013756469 |
| AC005592.2 | 3.1256451 | 3.1863841 | 0.836833 | 0.071337 | 0.107591269 |
| RP11-462L8.1 | 2.0153951 | 1.2147061 | 1.409994 | 0.051938432 | 0.241660192 |
| RP11-804A23.2 | 2.6748124 | 1.3870401 | 0.716205 | -0.014106491 | 0.750633967 |
| LINC01021 | 2.6710144 | 4.5648531 | 0.123282 | -0.01865195 | 0.67432232 |
| RP11-91P24.6 | 2.9223842 | 1.1548778 | 0.382899 | -0.068478 | 0.122474488 |
| LINC00659 | 2.9096405 | 6.0962749 | 0.157958 | -0.075717 | 0.087602432 |
| TM4SF1-AS1 | 5.9585665 | 1.8049045 | 0.131512 | -0.149827 | 0.000687659 |
| RP11-57A19.2 | 2.4947207 | 2.1922369 | 0.111283 | -0.22328045 | 3.50E-07 |
| LINC01273 | 2.9081375 | 1.6004791 | 0.34261 | -0.269889292 | 5.82E-10 |
| LINC01356 | 5.7408823 | 2.5437845 | 0.222671 | -0.358877 | 6.01E-17 |
| MAFG-AS1 | 3.439912 | 2.9981708 | 0.134927 | -0.40096 | 4.05E-21 |
| FOXP4-AS1 | 4.9253356 | 2.3858744 | 0.158978 | -0.453298 | 3.28E-27 |
| SNHG11 | 2.0436676 | 1.2115239 | 0.137562 | -0.656651492 | 3.10E-64 |

# Table S6. Mass spectrometry protein identification results for biotinylated LUCAT1 RNA pull down

| Proteins | HCT-116 | | | RKO | | |
|---|---|---|---|---|---|---|
| | PepCount. | Unique PepCount | Cover Percent | PepCount | Unique PepCount | Cover Percent |
| PTBP1 | 39 | 17 | 32.07% | 46 | 22 | 41.75% |
| STAU1 | 10 | 10 | 22.27% | 4 | 4 | 6.69% |
| TF | 9 | 8 | 14.33% | 1 | 1 | 1.18% |
| ATP5A1 | 5 | 5 | 7.78% | 18 | 15 | 26.04% |
| EEF1A1 | 3 | 3 | 10.36% | 5 | 5 | 10.82% |
| EEF1A1P5 | 3 | 3 | 5.63% | 5 | 5 | 10.82% |
| PDIA3 | 2 | 2 | 4.16% | 6 | 6 | 11.68% |
| JUP | 2 | 2 | 2.95% | 3 | 3 | 3.22% |
| HSPD1 | 2 | 2 | 2.97% | 2 | 2 | 2.62% |
| CFI | 2 | 2 | 3.17% | 1 | 1 | 1.02% |
| DARS | 1 | 1 | 5.32% | 9 | 9 | 18.16% |
| FLG | 1 | 1 | 0.50% | 5 | 3 | 0.65% |
| FLG2 | 1 | 1 | 0.50% | 3 | 3 | 1.38% |
| DLD | 1 | 1 | 1.44% | 2 | 2 | 3.34% |
| RPS27A | 1 | 1 | 3.85% | 2 | 2 | 11.54% |
| SYNE1 | 1 | 1 | 0.07% | 2 | 2 | 0.14% |
| UBA52 | 1 | 1 | 4.69% | 2 | 2 | 14.06% |
| UBB | 1 | 1 | 3.92% | 2 | 2 | 11.76% |
| UBC | 1 | 1 | 3.55% | 2 | 2 | 5.90% |
| ADGRE1 | 1 | 1 | 1.24% | 1 | 1 | 1.24% |
| ANXA3 | 1 | 1 | 1.55% | 1 | 1 | 1.76% |
| APBA2BP | 1 | 1 | 1.83% | 1 | 1 | 1.83% |
| BIN2 | 1 | 1 | 1.48% | 1 | 1 | 1.48% |
| BRWD3 | 1 | 1 | 0.39% | 1 | 1 | 0.33% |
| C3ORF33 | 1 | 1 | 2.04% | 1 | 1 | 2.04% |
| CARD4 | 1 | 1 | 0.52% | 1 | 1 | 0.52% |
| CCNI | 1 | 1 | 3.39% | 1 | 1 | 3.39% |
| CD3EAP | 2 | 1 | 1.40% | 3 | 1 | 1.53% |
| CDCA5 | 1 | 1 | 1.98% | 1 | 1 | 1.98% |
| CDH13 | 1 | 1 | 7.46% | 1 | 1 | 7.46% |
| CLK3 | 2 | 1 | 1.10% | 2 | 1 | 1.10% |
| CSF2RB | 5 | 1 | 7.41% | 5 | 1 | 7.41% |
| CYP4B1 | 1 | 1 | 1.37% | 1 | 1 | 1.41% |
| CYP7B1 | 1 | 1 | 1.02% | 1 | 1 | 1.02% |
| DDX46 | 1 | 1 | 11.43% | 1 | 1 | 0.68% |
| DLST | 1 | 1 | 1.77% | 1 | 1 | 1.77% |
| FABP5 | 1 | 1 | 6.67% | 1 | 1 | 8.91% |

| | | | | | | |
|---|---|---|---|---|---|---|
| FLJ00246 | 1 | 1 | 0.47% | 1 | 1 | 0.47% |
| GCC2 | 1 | 1 | 0.32% | 1 | 1 | 2.02% |
| GIMAP4 | 1 | 1 | 1.75% | 1 | 1 | 3.97% |
| GIMAP7 | 1 | 1 | 2.00% | 1 | 1 | 2.00% |
| GJA10 | 1 | 1 | 1.10% | 1 | 1 | 1.10% |
| GOLGB1 | 1 | 1 | 0.18% | 1 | 1 | 0.18% |
| HIAN7 | 1 | 1 | 2.00% | 1 | 1 | 2.00% |
| HIMAP4 | 1 | 1 | 1.82% | 1 | 1 | 1.82% |
| HS3ST6 | 2 | 1 | 2.63% | 2 | 1 | 2.63% |
| KCNAB1 | 2 | 1 | 1.61% | 3 | 1 | 1.61% |
| KDM2A | 1 | 1 | 0.52% | 1 | 1 | 0.52% |
| KLK13 | 1 | 1 | 8.16% | 2 | 1 | 8.16% |
| LMO2 | 1 | 1 | 3.80% | 1 | 1 | 3.80% |
| LOC392742 | 2 | 1 | 0.24% | 1 | 1 | 0.24% |
| LRRC8B | 1 | 1 | 0.62% | 1 | 1 | 0.62% |
| MBP | 1 | 1 | 3.82% | 1 | 1 | 1.97% |
| METTL2A | 1 | 1 | 1.32% | 1 | 1 | 1.60% |
| METTL2B | 1 | 1 | 1.32% | 1 | 1 | 1.32% |
| METTL8 | 1 | 1 | 1.32% | 1 | 1 | 2.33% |
| MLF2 | 2 | 1 | 4.03% | 1 | 1 | 4.03% |
| MLH3 | 1 | 1 | 0.48% | 1 | 1 | 0.49% |
| NECAB3 | 1 | 1 | 1.73% | 1 | 1 | 1.68% |
| NOD1 | 1 | 1 | 0.64% | 1 | 1 | 0.64% |
| OCLN | 1 | 1 | 1.72% | 1 | 1 | 1.72% |
| PCLO | 2 | 1 | 0.18% | 1 | 1 | 0.18% |
| PDS5B | 1 | 1 | 0.35% | 1 | 1 | 0.35% |
| PIBF1 | 1 | 1 | 0.66% | 1 | 1 | 0.66% |
| SHMT2 | 1 | 1 | 1.04% | 1 | 1 | 2.92% |
| SLC30A3 | 2 | 1 | 2.65% | 1 | 1 | 2.65% |
| SOGA3 | 1 | 1 | 0.84% | 1 | 1 | 0.85% |
| TCERG1 | 1 | 1 | 0.48% | 1 | 1 | 0.46% |
| TMPRSS13 | 1 | 1 | 1.37% | 1 | 1 | 1.37% |
| UHRF1BP1L | 1 | 1 | 0.54% | 1 | 1 | 0.54% |
| VN1R5 | 1 | 1 | 1.96% | 1 | 1 | 1.96% |
| XP32 | 1 | 1 | 3.20% | 2 | 1 | 3.20% |

# Table S7. Correlation of the clinicopathological features with tumor LUCAT1 expression in CRC

| Clinical Variables | P | Low LUCAT1 | | High LUCAT1 | | Total No. | Unknown No. |
|---|---|---|---|---|---|---|---|
| | | Case No. | (%) | Case No. | (%) | | |
| Age | 0.882 | | | | | 96 | 1 |
| >50 | | 12 | 32.4 | 20 | 33.9 | | |
| ≤50 | | 25 | 67.6 | 39 | 66.1 | | |
| Gender | 0.013 | | | | | 96 | 1 |
| Male | | 17 | 45.9 | 42 | 71.2 | | |
| Female | | 20 | 54.1 | 17 | 28.8 | | |
| TNM stage | 0.183 | | | | | 96 | 1 |
| T123 | | 12 | 32.4 | 12 | 20.3 | | |
| T4 | | 25 | 67.6 | 47 | 79.7 | | |
| Lympho node metastasis | 0.084 | | | | | 96 | 1 |
| Negative | | 23 | 62.2 | 26 | 44.1 | | |
| Positive | | 14 | 37.8 | 33 | 55.9 | | |
| Tumor volume | 0.866 | | | | | 95 | 2 |
| ≤5 | | 28 | 75.7 | 43 | 74.1 | | |
| >5 | | 9 | 24.3 | 15 | 25.9 | | |
| Histologic grade | 0.016 | | | | | 87 | 10 |
| G1/G2 | | 31 | 86.1 | 32 | 62.7 | | |
| G3 | | 5 | 13.9 | 19 | 37.3 | | |
| p21 | 0.046 | | | | | 94 | 3 |
| Positive | | 18 | 51.4 | 41 | 72.2 | | |
| Negative | | 19 | 48.6 | 16 | 27.8 | | |
| NM23 | 0.027 | | | | | 86 | 11 |
| Positive | | 1 | 2.9 | 42 | 80.8 | | |
| Negative | | 33 | 97.1 | 10 | 19.2 | | |

# Table S8. Sequences of primers used in this study

| Identifier | Sequences (5'-3') |
|---|---|
| LUCAT1_F | GGAATTCAATCAACACTCCACTCA |
| LUCAT1_R | CGGGATCCGTATCTGCCTTTTCAGG |
| PTBP1_Fe | TGCTCTGTGTGCCATGGACG |
| PTBP1_Re | CTAGATGGTGGACTTGGAGAAGG |
| PTBP1_Fi | GGAATTCGCCACCATGGACGGCATTGTCCCAGA |
| PTBP1_Ri | CGGGATCCCTAGATGGTGGACTTGGAGAAGG |
| shLUCAT1-1_F | CACCGCACCAGAGACCACAAATTCCTCAAGGGAATTTGTGGT CTCTGGTGCTTTTTT |
| shLUCAT1-1_R | AAACAAAAAAGCACCAGAGACCACAAATTCCCTTGAGGAAT TTGTGGTCTCTGGTGC |
| shLUCAT1-2_F | CACCGGTATTTCTGACTTGGCTTTCTCAAGGAAAGCCAAGTC AGAAATACCTTTTTT |
| shLUCAT1-2_R | AAACAAAAAAGGTATTTCTGACTTGGCTTTCCTTGAGAAAGC CAAGTCAGAAATACC |
| LUCAT1_A_F | GATCACTAATACGACTCACTATAGGaccacaaattcctctttg |
| LUCAT1_A_R | TTCAGCCCCTTTAGCAGTT |
| LUCAT1_B_F | GATCACTAATACGACTCACTATAGGtggtatttctgacttggc |
| LUCAT1_B_R | ATAGCAAACAGCAAGTTG |
| LUCAT1_C_F | GATCACTAATACGACTCACTATAGGgaacagctttcatcctct |
| LUCAT1_C_R | GTGTAGCATCAGGACAAA |
| LUCAT1_D_F | GATCACTAATACGACTCACTATAGGaatcaacactccactcag |
| LUCAT1_D_R | GTGACACTGAGCAAGGCC |
| LUCAT1_E_F | GATCACTAATACGACTCACTATAGGcttaccagctgtccctcagt |
| LUCAT1_E_R | GTATCTGCCTTTTCAGGCAG |
| PTBP1_NRRM1_F | GGAATTCGACGGCATTGTCCCAGA |
| PTBP1_NRRM1_R | CGGGATCCCTAGGCCGCCGCCGAGGCAGCC |
| PTBP1_RRM1_F | GGAATTCGACAGCCGAAGTGCAGGCGTCCCC |
| PTBP1_RRM1_R | CGGGATCCCTAGGCCGCCGCCGAGGCAGCC |
| PTBP1_RRM2_F | GGAATTCGGGAACCTGGCCTTGGCTGC |
| PTBP1_RRM2_R | CGGGATCCCTAGGGGGCCAGGGCGCCGTGGACG |
| PTBP1_RRM34_F | GGAATTCTCCGTTCCGAACGTCCACGG |
| PTBP1_RRM34_R | CGGGATCCCTAGATGGTGGACTTGGAGAAGG |
| HRE2_F | GGGGTACCGCTATAGAAAAGGGTGTGTCATCT |
| HRE2_R | CCGCTCGAGATCTCCCTGTGCTCAAGTGAT |
| shPTBP1_F | CACCGGACGGCATTGTCCCAGATATTCAAGATATCTGGGACA ATGCCGTCCTTTTTT |
| shPTBP1_R | AAACAAAAAAGGACGGCATTGTCCCAGATATCTTGAATATCT GGGACAATGCCGTCC |

## Table S9. Sequences of ChIP-qPCR primers to detect HREs

| Identifier | Forward Primer | Reverse Primer |
|---|---|---|
| Site 1 | AAGGCGTGCTGATTACTTGT | TCTCCCCAGTAGCTGAAATT |
| Site 2 | AACAAAACAATTCAGCTGGG | TCTCCCTGTGCTCAAGTGAT |
| Site 3 | CTAAAAATACAAATAGCCAGGTGTG | CCTAGATTTTGTTACTGAGACTGA |
| Site 4 | ATGGGCTACATGCTGAGCTA | CGTTGTGTACTGGAGTTTCC |
| Site 5 | GGTATATACAAGATGATGTTCTGG | TCCTCCCATAACCCTTTGAA |
| Site 6 | ACCGTGTTAGCCAGGATGGT | CACAGTGGCTCACGCCTGTA |
| Site 7 | TGTGCTATACATGCTGTTGA | ACCTTTTCATTGGGAGATGA |
| Site 8 | GTTGGCCTTATGTTAGCATC | CCATGTACACGTTCAATCTA |
| Site 9 | TTCACAGCTTGAGGTGGTTT | TTGCAAATAACACACGTTCG |

## Table S10. Antibodies used in this study

| Identifier | Catalog Number | Company |
|---|---|---|
| H2AX (Phospho-Ser139) | D155127 | Sangon |
| H2AX | 10856-1-AP | Proteintech |
| PTBP1 | 12582-1-AP | Proteintech |
| STAU1 | 14225-1-AP | Proteintech |
| HIF1A | 14179 | Cell Signaling |
| FLAG M2 | F1804 | Sigma |
| β-actin | 60008-1-Ig | Proteintech |
| Goat anti-Mouse IgG (H+L), HRP conjugate | SA00001-1 | Proteintech |
| Goat anti-Rabbit IgG (H+L), HRP conjugate | SA00001-2 | Proteintech |
| VeriBlot for IP secondary antibody | ab131366 | Abcam |
| PCNA | 10205-2-AP | Proteintech |
